# Specific Lipidomic Shifts in Chronic Lymphocytic Leukemia at Diagnosis

**DOI:** 10.3390/cancers18060896

**Published:** 2026-03-10

**Authors:** Julia Wojnicka, Michał Kiełbus, Paulina Mertowska, Sebastian Mertowski, Ewelina Grywalska, Piotr Sosnowski, Alicja Wielgosz, Anna Kozub-Pędrak, Barbara Sosnowska-Pasiarska, Maria Klatka, Janusz Klatka, Anna Błażewicz

**Affiliations:** 1 Department of Pathobiochemistry and Interdisciplinary Applications of Ion Chromatography, Medical University of Lublin, 1 Chodźki St., 20-093 Lublin, Poland; julia.wojnicka@umlub.pl; 2 Department of Biochemistry and Molecular Biology, Medical University of Lublin, 1 Chodźki St., 20-093 Lublin, Poland; michal.kielbus@umlub.pl; 3 Department of Experimental Immunology, Medical University of Lublin, 4a Chodźki St., 20-093 Lublin, Poland; paulina.mertowska@umlub.pl (P.M.); sebastian.mertowski@umlub.pl (S.M.); ewelina.grywalska@umlub.pl (E.G.); 4 Department of Bioanalytics, Medical University of Lublin, 8b Jaczewskiego St., 20-090 Lublin, Polandalicja.wielgosz@umlub.pl (A.W.); annakozub@umlub.pl (A.K.-P.); 5 Department of Oncocardiology, Holy Cross Cancer Center, 25-734 Kielce, Poland; spbasia@gmail.com; 6 Department of Pediatric Endocrinology and Diabetology, Medical University of Lublin, 1 Gębali St., 20-093 Lublin, Poland; maria.klatka@umlub.pl; 7 Department of Otolaryngology and Laryngological Oncology, Medical University of Lublin, 8 Jaczewskiego St., 20-954 Lublin, Poland; janusz.klatka@umlub.pl

**Keywords:** chronic lymphocytic leukemia (CLL), metabolic reprogramming, carnitines, ether-linked phospholipids, lipidomics

## Abstract

Background: Chronic lymphocytic leukemia (CLL) is the most common adult leukemia and is characterized by dysregulated apoptosis and metabolic reprogramming, including alterations in lipid metabolism. However, the plasma lipidome of newly diagnosed, treatment-naïve CLL patients remains insufficiently characterized. This study aimed to define disease-specific plasma lipidomic alterations, identify discriminatory lipid species, and investigate associated metabolic pathways. Methods: The study cohort consisted of 41 participants (median age 75 years, range: 40–86), including 30 newly diagnosed, treatment-naïve CLL patients (median age 75 years, range: 40–86) and 11 age- and sex-matched healthy controls (median age 75 years, range: 41–85). Targeted lipidomic profiling was performed on plasma samples using liquid chromatography with tandem mass spectrometry (LC-MS/MS). Data processing was conducted in R using LipidSigR. Statistical analyses employed the Wilcoxon–Mann–Whitney test with Benjamini–Hochberg correction. To address data dimensionality, Boruta machine learning and pathway enrichment analyses were applied. Gene–lipid associations were further explored using GATOm, followed by Metascape analysis to identify enriched biological processes. Results: A total of 124 lipid species from five major classes (phosphatidylcholines, lysophosphatidylcholines, sphingomyelins, ether-linked phosphatidylcholines, and acylcarnitines) were quantified. CLL patients exhibited significant enrichment of acylcarnitines, saturated phosphatidylcholines, and sphingolipids compared with controls. Principal component analysis showed partial separation by disease status. Machine learning identified carnitines and ether-linked phospholipids as key discriminators. Integrated gene–lipid analyses revealed significant enrichment of lipid metabolism-related pathways, particularly glycerolipid and phosphatidylcholine metabolism, as well as lipid catabolism, ether lipid metabolism, and fatty acid metabolism. Conclusions: Treatment-naïve CLL patients display distinct plasma lipidomic signatures indicative of disease-specific metabolic reprogramming. Integrated lipidomic and predictive pathway analyses suggest disruptions in lipid metabolic pathways and highlight carnitines and ether-linked phospholipids as biological markers warranting further investigation as potential CLL biomarkers.

## Simple Summary

Chronic lymphocytic leukemia (CLL) is a common type of adult blood cancer in which cells survive longer than normal, partly due to changes in how they process fats and other molecules for energy. This study examined the blood plasma of newly diagnosed patients who had not yet received treatment to identify unique patterns in lipid molecules. We found that patients with CLL had higher levels of certain fats, including carnitines and specific phospholipids, compared with healthy individuals. By analyzing these lipid changes using predictive bioinformatics tools, we identified that several pathways involved in lipid metabolism are likely disrupted. These findings improve our understanding of how this disease alters the body’s metabolism and could inform future research on biomarkers for earlier disease detection and treatment development.

## 1. Introduction

Chronic lymphocytic leukemia (CLL) is the most common leukemia in adults [1], characterized by the accumulation of mature clonal B lymphocytes. The 5-year survival rate for CLL is currently ~88–89%, whereas the 10-year survival rate is lower, but still quite high in many patient groups [2]. CLL pathogenesis involves genetic alterations, aberrant apoptosis, and dysregulated signaling through pathways such as BCR, PI3K/AKT/mTOR, and NF-κB. The tumor microenvironment and immune dysfunction contribute to disease persistence and progression [3]. A study by Kosmaczewska et al. [4] suggested that dysregulated T-cell turnover, driven by abnormal expression of G1-phase regulators, contributes to the pathogenesis of CLL and may serve as a prognostic indicator. Building on this, Stokłosa has emphasized that interactions between T cells and CLL cells within the tumor microenvironment may significantly influence disease progression [5]. While diagnosis and risk stratification largely rely on genetic markers, the growing interest in metabolic reprogramming, particularly lipid metabolism, offers promising new insights into the pathobiology of CLL and potential biomarkers [6]. Advances in lipidomics have revealed significant changes in lipid metabolism in various cancers, including acute myeloid leukemia (AML), as shown in a study of a small group of 20 patients conducted by Pabst et al. [7].

Lipid profile abnormalities, including cholesterol fractions alone, have been also reported in patients with CLL, although results are inconsistent [6,8]. Some studies have shown that CLL cells undergo significant lipid metabolic reprogramming, playing a significant role in the initiation and progression of CLL [9]. Overexpression of lipoprotein lipase, a marker associated with poor prognosis in CLL, along with increased fatty acid oxidation and altered cholesterol processing, has been reported in CLL and contributes to leukemic cell survival, immune evasion, and therapy resistance [10,11,12,13]. Recent studies by Lu et al. demonstrated a previously unidentified role and mechanism of ectonucleotide pyrophosphatase/phosphodiesterase 2 in the regulation of lipid metabolism in CLL [14]. However, comprehensive lipidomic profiling at the time of CLL diagnosis is still lacking. Moreover, no study conducted exclusively in a Polish population of patients with CLL has been published, even though chronic lymphocytic leukemia is the most common hematologic malignancy in Poland [15]. Currently, there are over 10,000 people living with this disease in Poland, but in some patients the disease may progress very slowly, without symptoms for a long time, so these figures are underestimated. Variability in the clinical course of CLL necessitates individualized treatment approaches to ensure optimal survival outcomes alongside an acceptable safety profile, especially for patients with unfavorable prognostic characteristics [16]. In addition to genetic and immunochemical determinants of disease progression, metabolic reprogramming, including alterations in lipid metabolism, has been increasingly recognized as a feature of CLL cell biology.

To better understand the metabolic changes present at disease onset, we examined a broad lipidomic profile of blood plasma from newly diagnosed CLL patients before treatment, focusing on lipid classes involved in energy metabolism, membrane structure, and cell signaling. An age- and sex-matched healthy control group was included to attribute lipid changes specifically to CLL rather than to age-related metabolic factors.

## 2. Materials and Methods

The study was approved by the Ethics Committee of the Medical University of Lublin (KE-0254/58/03/2024). The planning, conduct, and reporting of human research were in accordance with the Helsinki Declaration. The informed consent has been received.

### 2.1. Patient Characteristics

The overall study population comprised 41 participants with a median age of 75 years (range: 40–86). Specifically, the cohort included 30 newly diagnosed, treatment-naïve patients with CLL (median age 75 years, range: 40–86) and a control group of 11 healthy volunteers (median age 75 years, range: 41–85). Participants were recruited by the Department of Hematology and Bone Marrow Transplantation at the Świętokrzyskie Oncology Centre in Kielce, Poland. The diagnosis of CLL was established in accordance with the guidelines of the International Workshop on CLL (iwCLL), covering diagnostic criteria, treatment indications, response evaluation, and supportive care management [17], as well as the updated diagnostic and therapeutic recommendations published in 2022 [18].

The diagnostic process included a comprehensive clinical evaluation consisting of medical history, physical examination, and assessment of performance status. Hematological analysis involved a complete blood count (CBC) with peripheral blood smear evaluation. Immunophenotypic characterization of B lymphocytes was performed in the hospital laboratory using a BD FACSLyric™ flow cytometer (BD Biosciences, San Jose, CA, USA). All patients demonstrated >5 × 10^9^/L monoclonal B lymphocytes. Immunophenotyping confirmed the presence of B cells expressing CD5^+^CD19^+^ and CD5^+^CD19^+^CD23^+^ phenotypes. Biochemical evaluation included the quantification of serum immunoglobulins (IgG, IgA, and IgM), lactate dehydrogenase (LDH) activity, and beta-2-microglobulin concentration. All patients underwent fluorescence in situ hybridization (FISH) to detect common chromosomal aberrations associated with CLL. The cytogenetic findings were as follows: isolated deletion of 13q14.3 [13q14.3 (D13S319)] in 8 patients, isolated trisomy 12 [12p11.1–q11.1] in 2 patients, isolated deletion of 11q22.3 [11q22.3 (ATM)] in 2 patients, co-occurrence of 13q14.3 deletion and trisomy 12 in 1 patient, and no detectable abnormalities (del(17p13.1), del(13q14.3), trisomy 12, or del(11q22.3)) in 17 patients. All patients were classified as Rai stage 0 at diagnosis, with no evidence of hepatomegaly or splenomegaly.

Patient diagnosis, clinical assessment, and detailed demographic, clinical, phenotypic, biochemical, and cytogenetic data are summarized in Supplementary Tables S1 and S2. Any isolated anemia and/or thrombocytopenia noted in Supplementary Table S2 was not considered CLL-related cytopenias contributing to Rai staging at diagnosis.

Exclusion criteria encompassed a history of infections occurring within the preceding two months, autoimmune or allergic diseases, ongoing or prior immunomodulatory therapy, blood transfusions, obesity (body mass index (BMI) ≥ 30), type 2 diabetes mellitus, metabolic syndrome, use of medications known to affect lipid metabolism, such as nicotinic acid and derivatives, bile acid sequestrants, fibrates, HMG-CoA reductase inhibitors (statins), lipid modifying agents in combination with other drugs, and other lipid modifying agents, e.g., PCSK9 inhibitors, omega-3-triglycerides including other esters and acids, policosanol, and magnesium pyridoxal 5-phosphate glutamate. Women using hormone replacement therapy and contraceptives were excluded. Individuals following special or restrictive diets (e.g., ketogenic, vegan, low-fat) were excluded to reduce the potential influence of nutritional confounders on lipidomic profiling. Subjects on a regular diet with moderate physical activity, as well as non-smokers and alcohol avoiders, were eligible for the study.

### 2.2. Targeted Lipidomic Analysis

Fasting morning blood samples were collected at diagnosis, prior to treatment initiation. Plasma (0.5 mL) was immediately isolated using standard procedures to ensure sample stability. All samples were prepared and analyzed identically. AbsoluteIDQ p180 kit (Biocrates Life Sciences AG, Innsbruck, Austria) was used for the targeted analysis of a total of 145 metabolites using liquid chromatography with tandem mass spectrometry (LC-MS/MS). Preparation of samples was performed following the protocols described in our previous papers [19,20] and lipidomic analyses were performed on an Agilent Infinity II 1290 High Performance Liquid Chromatography (HPLC) coupled to triple quadrupole mass spectrometer 6470 (TQ MS, Agilent Technologies, Santa Clara, CA, USA). Data acquisition was performed using MassHunter Acquisition B.10.0 (Agilent Technologies, USA). Data analysis was conducted with MetIDQ (Biocrates, Austria) and MassHunter Quantitative (Agilent Technologies, USA).

Lipid shorthand notation follows LIPID MAPS^®^ guidelines [21], whereas genes shorthand notation follows Human Genome Variation Society (HGVS) nomenclature guidelines [22].

### 2.3. Data Analysis

A comprehensive lipidomics was conducted to investigate differences in lipid profiles between samples from the patients with CLL diagnosis and healthy controls. Lipid annotation and classification were performed using LipidSigR ver 0.7.0 [23], an R-based pipeline employing the rgoslin package for annotation [24]. Principal Component Analysis (PCA), implemented within LipidSigR, was used to explore variability and potential clustering patterns between the CLL and control groups. Differential Expression (DE) analysis between groups was performed using the Wilcoxon–Mann–Whitney test, with Benjamini–Hochberg correction for multiple comparisons. Results were deemed significant with an adjusted *p*-value of less than 0.05 and a fold change (FC) greater than 1. Positive log2FC values indicated higher lipid abundance in CLL samples, whereas negative values represented higher abundance in healthy controls. Structural and biophysical lipid characteristics were annotated using the LipidSigR computational pipeline, which integrates the rgoslin nomenclature parser. The metric “Total FA” designates the cumulative carbon chain length and the total number of double bonds across all esterified acyl groups attached to the intact lipid backbone; it does not represent unesterified free fatty acids. Furthermore, membrane biophysical properties—including bilayer thickness, lateral diffusion, and intrinsic curvature—were not experimentally measured in vitro. Instead, these parameters were computationally inferred using the functional annotation framework embedded within LipidSigR. Following structural parsing, individual lipid species were cross-referenced against the internal LipidSigR database. Specific biophysical categories (e.g., high, average, low, or positive/neutral/negative curvature) were algorithmically assigned based on established empirical relationships tied to the lipid headgroup class and the steric properties of their esterified acyl chains, specifically the cumulative carbon length and the degree of unsaturation. The Boruta version 0.7.0 R package was used to identify key lipid features distinguishing CLL samples from controls [25]. To ensure robustness, Boruta was run 100 times on random subsets containing 70% of the data (training), and another 100 runs were performed on the full dataset (validation). The algorithm utilized the getImpFerns importance measure and a significance threshold of *p* < 0.01. Features that were consistently selected as important in both conditions (appearing in more than 50 of the 100 runs in each case) were deemed stable. The validity of selected features was determined by calculating signal-to-noise ratios, expressed as the quotient of the feature’s median importance to the maximum (Ratio_to_ShadowMax) and mean (Ratio_to_ShadowMean) importance scores of the permuted shadow attributes. Features were ranked according to their medianImp values. Receiver Operating Characteristic (ROC) curve analysis was performed using the pROC package to evaluate the diagnostic capacity of selected lipid biomarkers in distinguishing CLL patients from healthy controls. Diagnostic performance was quantified by calculating the Area Under the Curve (AUC) with 95% confidence intervals. The optimal classification threshold for each lipid, along with its corresponding sensitivity and specificity, was determined by maximizing Youden’s J statistic. To associate identified lipids with corresponding genes, the Gene Annotation Tool for Metabolomics (GATOm) [26] within LipidSigR was used. Gene–lipid interaction networks were constructed from GATOm pathway analysis results. Reaction paths were bipartite-mapped to extract direct associations between specific genes and individual lipid species. Uninformative generic nodes (e.g., Carnicor) were filtered prior to graph construction to reduce topological noise. Network visualization was executed in R using the igraph and ggraph packages, applying the force-directed Kamada–Kawai layout algorithm. Node sizes were scaled by degree centrality to emphasize highly connected metabolic hubs. Genes derived from GATOm analysis underwent functional enrichment analysis with Metascape online tool [27], identifying biological processes and lipid metabolism pathways significantly associated with CLL. All data visualizations and statistical analyses were performed using the R programming environment (version 4.1.3).

## 3. Results

**Figure 1:**
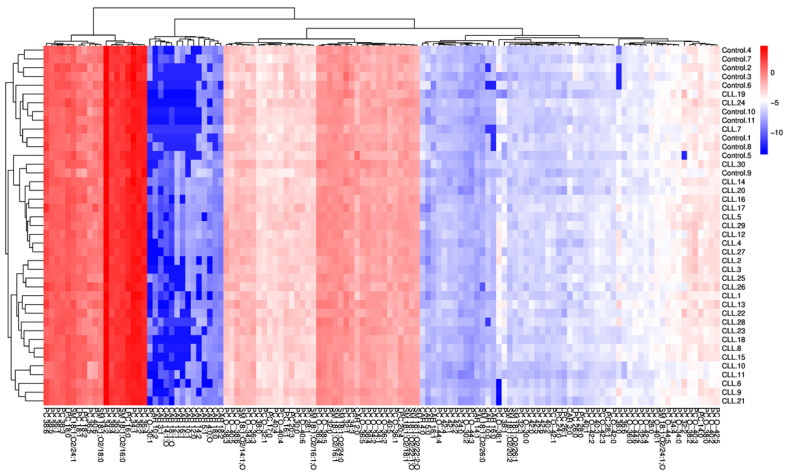
Heatmap of log-transformed normalized abundances of selected lipids in the entire study cohort. The analysis includes all 41 participants (30 newly diagnosed, treatment-naïve CLL patients and 11 healthy controls). Columns represent individual samples, and rows represent the 124 identified lipid species. Unsupervised hierarchical clustering was applied to both samples and lipid features. The color scale indicates relative lipid expression levels, ranging from blue (lowest abundance) to red (highest abundance).

A total of 124 lipids, classified into five lipid classes—phosphatidylcholines (PC), lysophosphatidylcholines (LPC), sphingomyelins (SM), ether-linked phosphatidylcholines (PC O-), and carnitines (CAR)—were identified using the LipidSigR pipeline based on their structural characteristics. The heatmap illustrates the distribution of lipid profiles across all analyzed samples, highlighting the variations among different groups (Figure 1). This visualization highlights both differences and similarities between samples concerning lipid abundance and composition and enables the identification of lipid profiles associated with CLL and control groups. The distinct lipid expression patterns and partial aggregation of samples based on disease status are shown. This baseline variation in lipid composition demonstrates clear metabolic divergence between treatment-naïve CLL patients and healthy individuals.

To establish a foundational understanding of the baseline lipidomic alterations, basic descriptive and comparative statistics were evaluated prior to multidimensional modeling. The median abundances of aggregated lipid classes were compared between treatment-naïve CLL patients and healthy controls using the Wilcoxon–Mann–Whitney test with Benjamini–Hochberg correction for multiple testing. At the class level, significant metabolic dysregulation was restricted to three lipid categories. Lysophosphatidylcholines (LPC) and ether-linked phosphatidylcholines (PC O-) were significantly enriched in the CLL cohort (adj_*p* = 0.045 and adj_*p* < 0.001, respectively). Conversely, bulk phosphatidylcholines (PC) demonstrated a significant depletion in CLL patients compared to controls (adj_*p* < 0.001). The aggregated abundances of acylcarnitines (CAR) and sphingomyelins (SM) did not exhibit statistically significant differences between the groups. Comprehensive descriptive statistics, encompassing median abundances, interquartile ranges, pseudo fold changes, and adjusted *p*-values for all lipid classes and individual species, are detailed in Supplementary Table S3.

**Figure 2:**
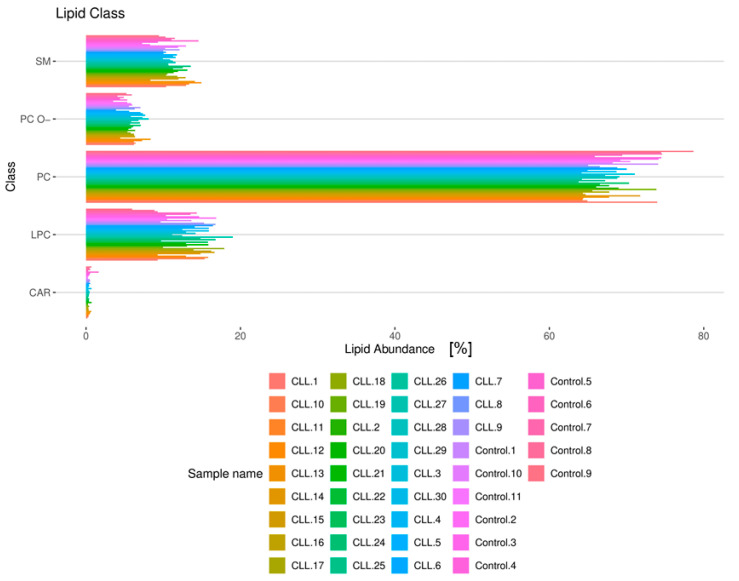
Lipid composition in CLL patients and healthy controls. The bar plot quantifies the absolute count and relative composition of the 124 detected lipids, categorized into five structural classes: phosphatidylcholines (PC), lysophosphatidylcholines (LPC), sphingomyelins (SM), ether-linked phosphatidylcholines (PC O-), and acylcarnitines (CAR). Data represent the aggregate lipidome architecture of the entire study cohort (30 treatment-naïve CLL patients and 11 healthy controls).

The most abundant lipid classes were PC, LPC, and SM, indicating their predominant presence in the analyzed samples. In contrast, PC O- and CAR were the least represented, suggesting a lower prevalence of these lipid types in the studied groups (Figure 2 and Figure S1A). Figure 2 presents a stacked bar plot illustrating lipid composition in CLL patients and healthy controls. Structural classification of the 124 quantified lipid species defined the baseline lipidome composition of the study cohort. PC constituted the most abundant lipid class, followed by LPC and SM, which formed the bulk of the circulating lipid pool. Conversely, PC O- and CAR represented minority fractions. This distribution establishes the structural framework required for subsequent differential expression analysis and highlights the dominance of glycerophospholipids in the plasma lipidome.

**Figure 3:**
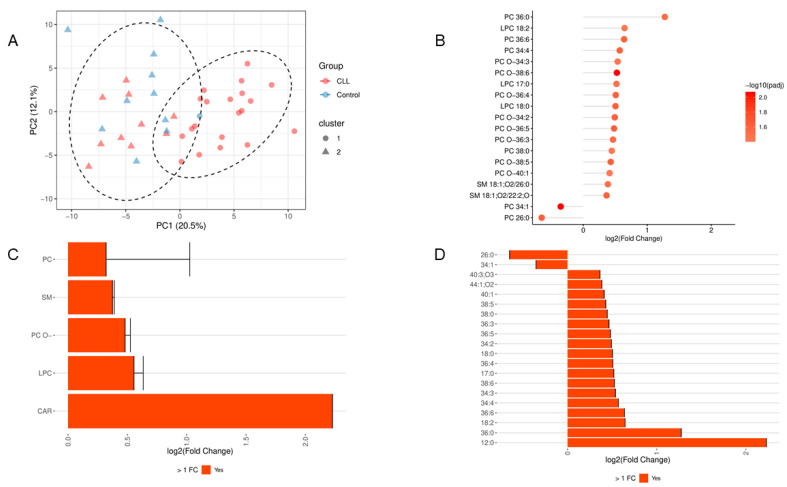
Principal component analysis and differential expression of the plasma lipidome in treatment-naïve CLL patients versus healthy controls. (**A**) Principal component analysis (PCA) score plot of log2-transformed lipid abundances. Ellipses represent 95% confidence intervals for each group. PC1 and PC2 account for 20.5% and 12.1% of the total variance, respectively. (**B**) Differential expression of individual lipid species. Positive log2(fold change) values indicate significant enrichment in the CLL cohort; negative values indicate depletion. (**C**) Aggregate differential expression stratified by primary lipid class. (**D**) Differential expression mapped by total fatty acid (Total FA) composition. Total FA denotes the combined number of carbon atoms and double bonds present in the esterified acyl chains of the intact complex lipids, not unesterified free fatty acids.

To determine if global lipidomic profiles differentiate disease states, principal component analysis (PCA) was executed (Figure 3A). The model demonstrated a partial spatial separation between CLL patients and healthy controls along the first two principal components (cumulative variance: 32.6%), indicating a distinct baseline metabolic shift driven by the malignancy. Subsequent DE analysis isolated the specific variables driving this separation. At the individual species level (Figure 3B), a systemic lipid upregulation was observed in CLL plasma. Significant enrichment was recorded for multiple species, dominated by CAR 12:0, PC 36:0, and LPC 18:2, whereas only PC 34:1 and PC 26:0 exhibited depletion relative to controls. When aggregated by structural class (Figure 3C), all analyzed categories demonstrated positive fold changes (enrichment in CLL). The magnitude of this shift was hierarchical: CAR exhibited the most severe dysregulation, followed sequentially by LPC, PC O−, SM, and PC. To resolve structural patterns within the complex lipids, differential expression was mapped against the total fatty acid (Total FA) metric (Figure 3D). Importantly, the “Total FA” parameter strictly represents the combined carbon chain length and number of double bonds of the esterified acyl chains attached to the intact lipid backbone; it does not refer to free fatty acids derived from hydrolysis. This analysis revealed that two Total FA configurations (26:0 and 34:1) exhibited negative log2(FC), indicating a relative depletion of lipids bearing these structural features in CLL compared to healthy controls. Conversely, a broad spectrum of Total FA configurations demonstrated positive log2(FC), signifying systemic enrichment. The magnitude of this enrichment increased progressively across the identified acyl configurations in the following order: 40:3;O3, 44:1;O2, 40:1, 38:5, 38:0, 36:3, 36:5, 34:2, 18:0, 36:4, 17:0, 38:6, 34:3, 34:4, 36:6, 18:2, and 36:0, culminating in the 12:0 configuration, which displayed the most pronounced structural upregulation in the CLL cohort.

We conducted DE analysis to compare lipid profiles between CLL samples and healthy controls, with controls as the reference group. A positive FC indicates lipid enrichment in CLL, while a negative FC suggests depletion in CLL or enrichment in controls. This approach enabled the identification of lipids that are significantly upregulated or downregulated in the disease state compared to the healthy baseline. The volcano plot shows the relationship between log2 FC and statistical significance, highlighting enriched and depleted lipids in CLL compared to controls. It is attached as Figure S1B. The results (Figure 3B) revealed a clear pattern of lipid enrichment, with a majority of lipids showing increased levels in CLL compared to controls. Specifically, the following lipids were significantly enriched in CLL: CAR 12:0, PC 36:0, LPC 18:2, PC 36:6, PC 34:4, PC O−34:3, PC O−38:6, LPC 17:0, PC O−36:4;2.0, LPC 18:0, PC O−34:2, PC O−36:5, PC O−36:3, PC 38:0, PC O−38:5, PC O−40:1, SM 18:1;O2/26:0, and SM 18:1;O2/22:2;O. This indicates that these lipids are significantly more abundant in CLL samples, suggesting metabolic alterations associated with the disease state. In contrast, only two lipids showed enrichment in healthy controls, indicating a decrease in CLL: PC 34:1 and PC 26:0. The full table of DE analysis, along with the filtered significant results, is attached as Supplementary Tables S4 and S5, respectively.

**Figure 4:**
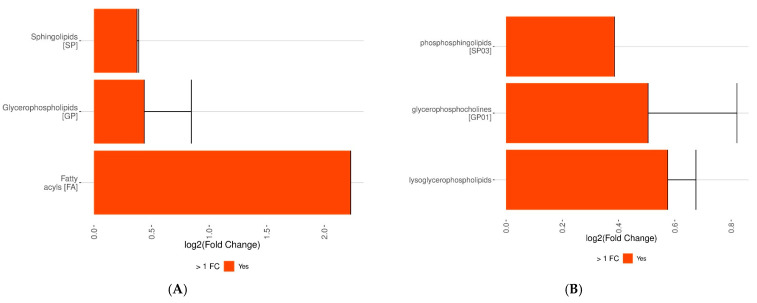

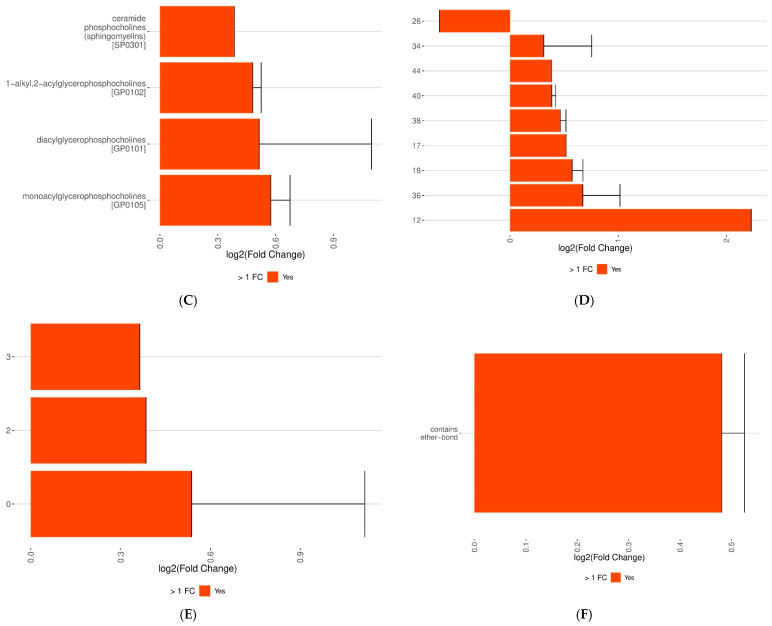
Lipid profiling and differential expression analysis between CLL and controls across different lipid groups. The enrichment of: the main lipid groups in the analyzed individuals (**A**), lipid subcategories (**B**), lipid subclasses (**C**), lipid subclasses based on the Total Carbon metric (**D**), lipid subclasses based on the Total -OH metric (**E**), lipid subclasses characterized by ether bonds (**F**).

The DE analysis comparing CLL to controls revealed a range of lipid classes (Figure 3C) with positive (FC), indicating enrichment in CLL. The smallest increase was observed for PC, followed by SM, PC O−, and LPC, while the highest enrichment was noted for CAR. This gradient suggests that carnitine metabolism is particularly upregulated in CLL. The differential expression analysis (Figure 3D) identified two Total Fatty Acids with negative (FC): 26:0 and 34:1, indicating that these fatty acids are depleted in CLL compared to healthy controls. In contrast, a range of Total FA showed positive FC, meaning they are enriched in CLL. Starting from the smallest positive FC to the largest, the identified fatty acids were: 40:3;O3, 44:1;O2, 40:1, 38:5, 38:0, 36:3, 36:5, 34:2, 18:0, 36:4, 17:0, 38:6, 34:3, 34:4, 36:6, 18:2, 36:0, and 12:0. This order reflects a gradual increase in enrichment, with 12:0 showing the highest positive FC among the analyzed fatty acids, suggesting a significant upregulation in CLL. The analysis revealed that all three lipid groups—sphingolipids, glycerophospholipids, and fatty acyls—showed a positive (FC), indicating higher levels in CLL compared to controls (Figure 4A). Among these, SP had the smallest increase, followed by GP, while FA exhibited the highest enrichment, suggesting that fatty acyl components are particularly elevated in CLL. It was also identified that three lipid subcategories are enriched in CLL compared to controls (Figure 4B). Among these, phosphosphingolipids (SP03) exhibited the smallest increase, followed by glycerophosphocholines (GP01), while lysoglycerophospholipids showed the highest enrichment. Moreover, a set of lipid subclasses with positive (FC) was identified, indicating enrichment in CLL compared to controls (Figure 4C). The smallest increase was observed for ceramide, followed by phosphocholines (sphingomyelins) (SP0301), then 1-alkyl,2-acylglycerophosphocholines (GP0102), and the highest enrichment was noted for diacylglycerophosphocholines (GP0101). Several lipid subclasses based on the Total Carbon (Total C) metric were identified (Figure 4D). A negative (FC) was observed for 26, indicating depletion in CLL compared to controls. In contrast, positive FC values were found for 34, 44, 40, 38, 17, 18, 36, and 12, indicating enrichment in CLL. This order reflects a gradual increase, with 12 showing the highest positive FC.

In Figure 4. a positive log2(fold change) indicates enrichment in CLL, while a negative log2(fold change) signifies enrichment in controls.

**Figure 5:**
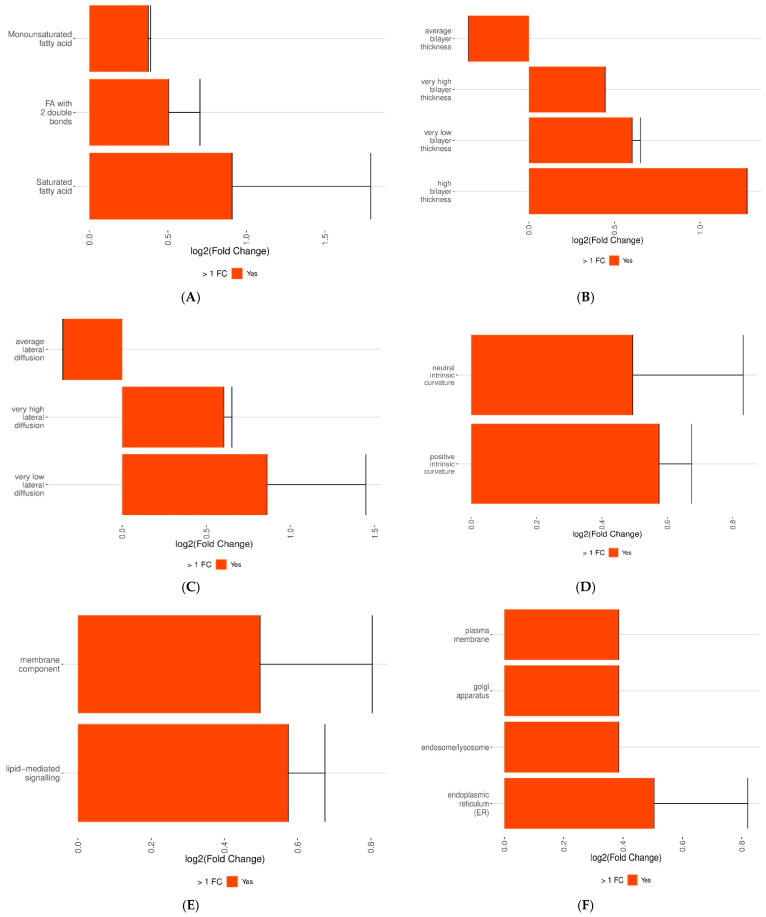
Lipid profiling and differential expression analysis between CLL and controls across various lipid metrics. Lipid subclass enrichment by various properties: (**A**) saturation level, (**B**) bilayer thickness, (**C**) lateral diffusion, (**D**) intrinsic curvature, (**E**) functional roles, and (**F**) associated cellular components.

Lipid subclasses based on the total OH metric were identified, all showing positive (FC), indicating enrichment in CLL compared to controls (Figure 4E). Total OH represents the number of hydroxyl groups present in lipid molecules, which influences their hydrophilicity. The FC value increases in the order of 3, 2, 0, with 0 showing the highest positive FC. This pattern suggests that lipids with fewer hydroxyl groups are particularly enriched in CLL, indicating a possible shift towards more hydrophobic lipid profiles in the disease state. Lipid subclasses characterized by the presence of ether bonds were identified, all showing positive FC, indicating enrichment in CLL compared to controls (Figure 4F). The ether bond metric reflects the presence of an oxygen atom linking two carbon atoms within the lipid structure, contributing to increased lipid stability and membrane rigidity. The FC values show a gradual increase, suggesting that lipids containing ether bonds are more prevalent in CLL, potentially reflecting altered membrane dynamics or signaling pathways associated with the disease. Lipid subclasses based on the degree of saturation were identified, all showing positive FC, indicating enrichment in CLL compared to controls (Figure 5A). FC values increase from monounsaturated fatty acids to fatty acids with two double bonds, with saturated fatty acids showing the highest positive FC. This suggests that saturated and less unsaturated fatty acids are enriched in CLL, possibly reflecting a shift toward more rigid, less fluid membranes.

In Figure 5 positive log2(fold change) indicates enrichment in CLL, whereas negative values indicate enrichment in controls. Lipid subclasses based on bilayer thickness showed varied FC (Figure 5B). Average bilayer thickness had a negative FC, indicating depletion in CLL. In contrast, positive FC values increased in the order: very high, very low, and high bilayer thickness, with high bilayer thickness showing the greatest enrichment in CLL. This suggests a shift towards more variable membrane thickness in the disease state. Lipid subclasses based on lateral diffusion displayed different FC patterns (Figure 5C). Average lateral diffusion exhibited a negative FC, indicating reduced levels in CLL compared to healthy controls. Conversely, positive FC values were observed in the following order: very high lateral diffusion and very low lateral diffusion, with the latter showing the greatest enrichment in CLL. This indicates that CLL is associated with altered lipid mobility within the membrane. Lipid subclasses based on intrinsic curvature showed positive (FC), indicating enrichment in CLL compared to controls (Figure 5D). The FC values increased in the order of neutral intrinsic curvature followed by positive intrinsic curvature, with positive intrinsic curvature showing the highest enrichment. This pattern suggests that CLL is associated with lipid profiles favoring curvature-promoting molecules, indicating potential changes in membrane flexibility and dynamics.

Lipid function and cellular component metrics showed higher abundance in CLL than controls. For function (Figure 5E), lipid-mediated signaling predominated over membrane components, indicating a shift toward signaling activity in CLL. In cellular components (Figure 5F), lipids associated with the plasma membrane were least abundant, with the ER showing the highest abundance in CLL. These changes suggest shifts in membrane structure and intracellular signaling, particularly in the ER, reflecting potential adaptations in lipid metabolism and membrane dynamics in CLL.

**Figure 6:**
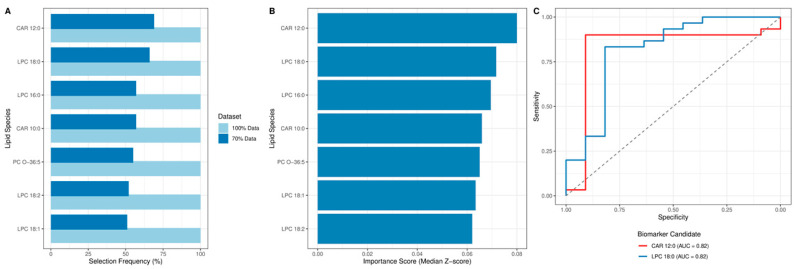
Identification and diagnostic evaluation of key lipid biomarkers in CLL. (**A**) Selection frequency of the stable discriminative lipid species identified by the Boruta feature selection algorithm. Bars indicate the frequency of confirmation across 100 iterations utilizing the full dataset (100% Data, blue) and bootstrap-sampled subsets (70% Data, light blue). (**B**) Feature importance of the confirmed lipids, expressed as the median Z-score (medianImp) derived from the Random Ferns algorithm. (**C**) Receiver Operating Characteristic (ROC) curves assessing the diagnostic capacity of the top-performing biomarker candidates. Diagnostic accuracy is quantified by the Area Under the Curve (AUC). The dashed line represents the performance of a random classifier (AUC = 0.5).

Machine learning analysis using the Boruta algorithm was performed to identify features distinguishing CLL from controls. The algorithm successfully selected lipids that consistently differentiate the two groups, both in the full dataset (all) and in the sampled subsets (sampled) (Figure 6A,B). Boruta analysis isolated seven stable lipid discriminators for CLL. Assessment of feature relevance established CAR 12:0 as the dominant variable (medianImp = 0.080), yielding an importance signal 25.38 times higher than the mean shadow attribute (Ratio_to_ShadowMean = 25.38). Successive high-ranking variables were LPC 18:0 (medianImp = 0.072; Ratio_to_ShadowMean = 22.74) and LPC 16:0 (medianImp = 0.070; Ratio_to_ShadowMean = 22.06). Remaining confirmed features (CAR 10:0, PC O-36:5, LPC 18:1, LPC 18:2) demonstrated medianImp values ranging from 0.062 to 0.066 and Ratio_to_ShadowMean values > 19.6. All identified lipids exhibited Ratio_to_ShadowMax values between 0.71 and 0.93, verifying robust signal separation from peak randomized noise (detailed in Supplementary Table S6). This result indicates that lipid metabolism, particularly involving carnitines and phosphatidylcholines, plays a key role in distinguishing CLL from controls, highlighting potential metabolic differences associated with the disease. The complete results of the feature selection analysis using the Boruta algorithm, identifying lipids that distinguish CLL patients from controls, are presented in Figure S2.

To isolate the most robust metabolic discriminators of CLL, the Boruta machine learning feature selection algorithm was applied. This dimensionality reduction identified seven stable lipid species that consistently differentiated CLL patients from healthy controls across both the full dataset and bootstrap-sampled subsets (Figure 6A). Assessment of the median importance scores (Z-scores) established CAR 12:0, LPC 18:0, and LPC 16:0 as the dominant variables driving the systemic separation (Figure 6B). To evaluate the clinical diagnostic potential of these defining metabolic alterations, Receiver Operating Characteristic (ROC) analysis was executed. Optimal biomarker candidates were selected based on rigid performance thresholds (AUC > 0.80, sensitivity > 0.70, specificity > 0.80) to minimize false-positive and false-negative classifications. The resulting ROC curves (Figure 6C) demonstrate the high diagnostic accuracy of CAR 12:0, PC O-34:2, and LPC 18:0, validating their utility as robust discriminant biomarkers for treatment-naïve CLL. A comprehensive summary of all evaluated diagnostic metrics, including optimal cutoff thresholds, sensitivity, specificity, and 95% confidence intervals for all seven candidate lipids, is provided in Supplementary Table S7.

**Figure 7:**
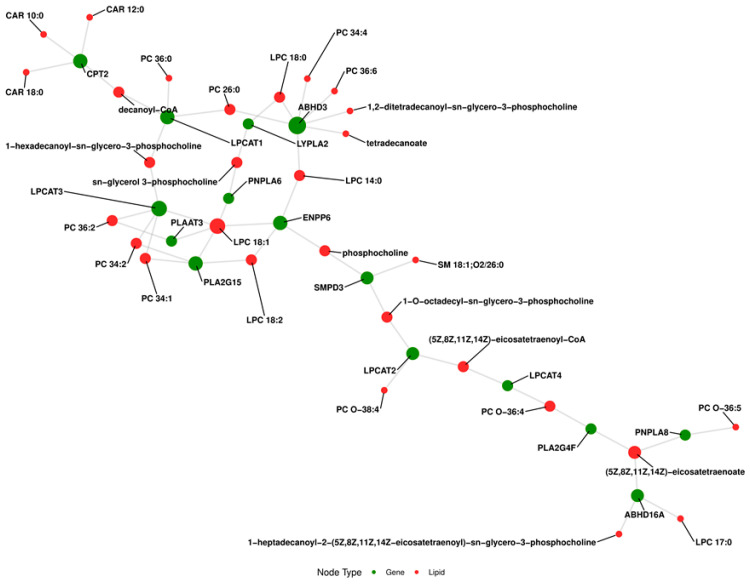
Schematic network of GATOm-derived gene–lipid interactions. The bipartite graph illustrates significant associations between regulatory genes (green nodes) and specific lipid molecules (red nodes) altered in chronic lymphocytic leukemia. Node size is proportional to degree centrality, highlighting highly interconnected metabolic hubs within the network. The spatial distribution of nodes was determined using the Kamada–Kawai layout algorithm.

To compare CLL samples with controls, we performed an analysis using GATOm, a tool designed to link genes to lipid metabolism pathways. The Supplementary Tables S8 and S9 provide detailed information on the genes predicted to encode enzymes responsible for these lipid alterations: ABHD3, CPT2, LPCAT3, PNPLA6, ENPP6, PLA2G4F, LPCAT4, LPCAT2, LPCAT1, SMPD3, PLA2G15, LYPLA2, PLAAT3, PNPLA8, ABHD16A. The first table contains gene functions, while the second lists associated lipids for each gene. Figure 7 and Supplementary Table S10 show the most significantly enriched biological processes related to lipid metabolism. A bipartite network analysis of GATOm enrichment mapped the predicted topological interactions between lipid-metabolizing enzymes and the differentially expressed lipid species. This schematic highlights potential regulatory hubs and visualizes the putative mechanistic links that may drive the observed lipidomic shifts in CLL. The Metascape analysis was performed to identify biological processes and pathways associated with the genes predicted by the GATOm network. The analysis revealed that the most significantly enriched biological processes are related to lipid metabolism, particularly the glycerolipid metabolic process and the phosphatidylcholine metabolic process, both showing high gene counts and substantial representation among the identified genes. Additionally, the metabolism of lipids pathway [28] was significantly enriched, indicating a strong link between these genes and lipid metabolic activities. Other relevant pathways include the lipid catabolic process, ether lipid metabolism, and fatty acid metabolic process, highlighting the role of lipid processing and remodeling in CLL.

## 4. Discussion

Our study identified statistically significant elevations in most lipid species across various lipid classes in CLL patients at diagnosis. To our knowledge, this is among the first comprehensive assessments of acylcarnitines, glycerophospholipids, and sphingolipids in untreated CLL patients. While lipid disturbances are well-documented in acute lymphoblastic leukemia, primarily as treatment-related effects [29], our research shifts the focus to intrinsic metabolic alterations in CLL. Previous studies have concentrated on traditional lipid markers such as total cholesterol, high-density lipoprotein cholesterol, and low-density lipoprotein cholesterol, primarily assessing their levels in response to therapy. In contrast, our study provides comprehensive lipidomic profiling at diagnosis, revealing important metabolic alterations in CLL.

Elevated levels of acylcarnitines, glycerophospholipids, and sphingolipids may reflect disease pathogenesis and progression. To date, only two studies have reported elevated acylcarnitine levels in patients with CLL compared to healthy individuals [29,30]. Despite certain methodological differences our results are consistent with these studies. Acylcarnitines facilitate long-chain fatty acid transport into mitochondria for β-oxidation, a crucial ATP production process [31]. Our findings of elevated medium-chain acylcarnitines (e.g., CAR 12:0, CAR 10:0) align with previous studies, suggesting increased metabolic flexibility and potential mitochondrial dysfunction in CLL cells. This may support the Warburg effect, where cancer cells rely on alternative energy pathways to meet heightened demands, contributing to survival and proliferation [32,33]. Targeting key pathways involved in acylcarnitine synthesis or its transport into mitochondria could offer a potential therapeutic strategy to impair CLL cell growth and survival.

To the best of our knowledge, there are no reports describing glycerophospholipid levels in samples derived from patients with CLL. The only relevant study identified in this context reports a fourfold increase in phosphatidylcholine levels within the non-histone protein fractions of chromosomal extracts from leukemic B lymphocytes [34]. Glycerophospholipids, essential components of cellular membranes, exhibited increased levels of saturated species (e.g., PC 36:0, PC 34:4, PC 36:6, PC 38:0). This shift towards more rigid membranes may facilitate cancer cell proliferation and survival. Additionally, alterations in phosphatidylinositol metabolism could activate the PI3K/AKT/mTOR signaling pathway, commonly hyperactivated in cancer cells, promoting growth and survival [35,36]. Lysophospholipids have been associated with chronic inflammation and oxidative stress. Elevated levels of LPC 18:2, LPC 17:0, and LPC 18:0 may further indicate increased phospholipase activity in response to oxidative stress, potentially contributing to membrane remodeling and supporting cancer cell proliferation. In CLL, where elevated levels of reactive oxygen species are present, alterations in lysophospholipids levels may reflect a compensatory response to oxidative stress and could play a role in disease progression [37]. The increased levels of lipids containing ether bonds—such as PC O-34:3, PC O-38:5, PC O-38:6, PC O-36:4;2.0, PC O-40:1, PC O-34:2, PC O-36:5, and PC O-36:3—which are known to confer greater stability and rigidity to cellular membranes [38]—may contribute to altered membrane dynamics in CLL. This is further supported by analyses of membrane biophysical properties, including reduced average bilayer thickness and decreased lateral diffusion. Regarding subcellular localization, the highest abundance of these lipids was observed in ER, potentially reflecting enhanced lipid synthesis and modification processes, as well as their role in cellular stress responses. Predictive functional analysis based on the altered lipid profiles revealed potential enrichment in pathways involved in glycerolipid and phosphatidylcholine metabolism. This implicates enzymes encoded by genes such as ABHD3, LPCAT3, and PNPLA8, suggesting a significant dysregulation of these metabolic processes in CLL. Nguyen Van Long et al. [39] demonstrated that elevated glucosylceramide C16:0 levels may serve as a prognostic marker in CLL. Jaeger et al. [40] further highlighted the significance of the ceramide-to-glucosylceramide ratio in predicting treatment response. Piszcz et al. [30] reported decreased levels of sphinganine, sphingosine phosphates, and hydroxysphingosine, indicating additional disruptions in sphingolipid metabolism. Hammadi et al. [41] showed that stabilizing sphingolipid levels can influence treatment efficacy. Their relevance as biomarkers is further supported by studies tracking sphingolipid levels in emerging therapies [42]. Thurgood et al. [43] found that CLL patients had lower SM 18:1/16:0 and higher SM 18:1/22:0 ratios, along with reduced ceramide 18:1/16:0 and 18:1/24:1, and lactosylceramide 18:1/20:0, while glucosylceramides 18:1/16:0 and 18:1/24:1 were significantly increased. In our study, although increases in sphingolipid levels were the smallest among studied lipid classes, they remained evident (e.g., SM 18:1;O2/26:0, SM 18:1;O2/22:2;O). Sphingolipids, including sphingosine-1-phosphate (S1P), regulate apoptosis and cell signaling [44]. Elevated levels may indicate dysregulated apoptotic pathways or a pro-inflammatory tumor microenvironment, supporting tumor growth. Our analysis showed elevated sphingomyelins and glucosylceramides, indicating altered sphingolipid metabolism in CLL, consistent with the mentioned previous findings.

### Advantages and Limitations of the Study

In this study, lipidomic analyses were restricted to blood plasma due to the greater accessibility of samples. Through careful experimental design, the use of appropriate internal controls, robust data preprocessing, and rigorous quality control procedures, we mitigated biological heterogeneity in plasma, matrix effects inherent to LC-MS/MS analyses, and instrument-related variability, which are recognized contributors to measured analytical noise in lipidomic data.

The present study focuses on patients at the time of CLL diagnosis, as this stage most accurately reflects the disease’s biological characteristics before therapeutic intervention and treatment-driven clonal evolution. In Poland, frontline CLL therapy currently follows three approaches: time-limited non-chemotherapy regimens, continuous targeted therapies, and time-limited immunochemotherapy. Collecting baseline data prior to treatment allows for the evaluation of potential diagnostic and prognostic biomarkers, correlations with established clinical and molecular risk factors, and a deeper understanding of early disease heterogeneity. In this biological and clinical context, our findings reveal key lipidomic alterations in CLL with significant implications for both research and therapy. Although genetic alterations and chromosomal aberrations have been extensively studied in CLL and remain central to prognostic stratification, disease progression and treatment response are known to be influenced by a complex interplay of genetic and non-genetic factors [45]. Lu et al.’s studies [46] demonstrated that CLL cells are characterized by metabolic heterogeneity, particularly in glycolysis and oxidative phosphorylation, that is influenced by genetic variants and may be associated with variable sensitivity to therapeutic compounds. The results of a plasma lipidome study in patients with chronic lymphocytic leukemia highlight the importance of broad biological and chemical characterization of molecules in understanding disease heterogeneity and subsequent response to treatment.

Altered ether lipid metabolism may suggest altered CLL cell survival and chemoresistance, while enrichment in saturated fatty acids suggests increased cell membrane stiffness, potentially modulating signaling pathways. By uncovering these changes, our study opens new avenues for mechanistic research and the development of targeted therapies for CLL. Lipid metabolism profiling may help identify patients most likely to benefit from such targeted interventions, supporting a personalized medical approach to CLL treatment.

The main limitation of our study is the relatively small sample size, which may reduce statistical power, limit generalizability, and constrain the identification of outliers. Nevertheless, this work provides valuable methodological insights, helps refine analytical procedures, and informs the design of future studies, ultimately supporting more robust evaluation of biomarkers and disease mechanisms in CLL. Future investigations must validate these exploratory findings in larger, independent patient cohorts to establish the clinical reliability of the identified lipid signatures. Subsequent research should longitudinally evaluate these specific lipid species, particularly acylcarnitines and ether-linked phospholipids, as prognostic markers, correlating their baseline abundance with treatment response and long-term clinical outcomes. Furthermore, integrating single-cell transcriptomic and lipidomic approaches is required to delineate the cell-intrinsic gene regulatory mechanisms and tumor microenvironment interactions driving this specific metabolic reprogramming in CLL.

This study is limited by the imbalance between CLL patients and controls, which may reduce statistical power and affect the stability of machine-learning–based feature selection. Although the Boruta algorithm aims to reduce random feature selection, its use in small datasets may increase the risk of overfitting and optimistic bias. Furthermore, the lack of an independent external validation cohort limits the evaluation of reproducibility and generalizability. Therefore, the identified features should be considered preliminary candidates requiring confirmation in larger, prospectively validated cohorts.

Our study was restricted to Rai stage 0 patients to reduce heterogeneity related to disease stage and treatment-related confounding factors. While this design improves cohort homogeneity, it inherently limits the generalizability of the findings to patients with more advanced CLL. Therefore, extrapolation to the broader CLL population should be performed with caution, and validation across different disease stages is warranted.

Finally, immunoglobulin heavy chain variable region (*IGHV*) gene mutation status was not available at the time of diagnosis for the patients included in this study, preventing stratified analyses according to this important prognostic marker. Consequently, the identified features should be considered preliminary candidates requiring confirmation in larger, prospectively validated cohorts.

## 5. Conclusions

In summary, our comprehensive lipidomic analysis performed before treatment initiation reveals distinct metabolic alterations of specific lipid molecules in patients with CLL. The enrichment of pathways related to glycerophospholipid and fatty acid metabolism, particularly those localized in the endoplasmic reticulum, indicates substantial reprogramming of lipid metabolism in CLL. These changes may reflect adaptive mechanisms of cancer cells associated with the maintenance of membrane homeostasis, endoplasmic reticulum stress, and the regulation of signaling processes. Based on in silico pathway mapping, we highlight a potential association between the observed lipid metabolic activity and enzymes encoded by specific genes: ABHD3, CPT2, LPCAT3, PNPLA6, ENPP6, PLA2G4F, LPCAT4, LPCAT2, LPCAT1, SMPD3, PLA2G15, LYPLA2, PLAAT3, PNPLA8, and ABHD16A, though their direct expression levels remain to be experimentally validated.

## Data Availability

The original contributions presented in this study are included in the article and in the Supplementary Materials. Further inquiries can be directed to the corresponding author.

## Supplementary Materials

The following supporting information can be downloaded at: https://www.mdpi.com/article/10.3390/cancers18060896/s1, Figure S1: Feature selection analysis using the Boruta algorithm identified lipids that distinguish CLL patients from controls; Figure S2: Lipid composition and differential expression analysis between CLL and controls; Table S1: Summary of clinical, phenotypic, biochemical and cytogenetic data; Table S2: Summary table of demographic, clinical and biochemical data of all study subjects (patients and controls); Table S3: Descriptive statistics and comparative analysis of aggregated lipid class abundances between treatment-naïve CLL patients and healthy controls; Table S4: Differential expression analysis; Table S5: The filtered significant results; Table S6: Feature importance and signal-to-noise metrics of the stable lipid discriminators identified by the Boruta algorithm; Table S7: Receiver Operating Characteristic (ROC) analysis metrics for candidate lipid biomarkers in chronic lymphocytic leukemia; Table S8: Gene functions (GATOm- Gene Annotation Tool for Metabolomics); Table S9: The associated lipids for each gene (GATOm); Table S10: The most significantly enriched biological processes related to lipid metabolism.

## Abbreviations

The following abbreviations are used in this manuscript:

AKT	Protein Kinase B
AML	Acute Myeloid Leukemia
AUC	Area Under the Curve
BCR	B-Cell Receptor
BMI	Body Mass Index
CAR	Carnitines
CBC	Complete Blood Count
CLL	Chronic Lymphocytic Leukemia
DE	Differential Expression Analysis
FC	Fold Change
GATOm	Gene Annotation Tool for Metabolomics
HGVS	Human Genome Variation Society
HPLC	High Performance Liquid Chromatography
IgG, IgA, IgM	Immunoglobulin G, Immunoglobulin A, Immunoglobulin M
IGHV	Immunoglobulin Heavy Chain Variable Region
LC-MS/MS	Liquid Chromatography Followed by Tandem Mass Spectrometry
LPC	Lysophosphatidylcholines
mTOR	Mammalian Target of Rapamycin
NF-κB	Nuclear Factor Kappa-Light-Chain-Enhancer of Activated B Cells
PC	Phosphatidylcholines
PC O-	Ether-Linked Phosphatidylcholines
PCA	Principal Component Analysis
PI3K	Phosphatidylinositol 3-Kinase
ROC	Receiver Operating Characteristic
S1P	Sphingosine-1-Phosphate
SM	Sphingomyelins
TQ	Triple Quadrupole
