# Supplementary material for: Specific Lipidomic Shifts in Chronic Lymphocytic Leukemia at Diagnosis
Source: Cancers (Basel). 2026 Mar 10;18(6):896. doi: 10.3390/cancers18060896 (PMC13024246; doi:10.3390/cancers18060896)
Supplement: Supplementary file 1 [file cancers-18-00896-s001.zip › cancers-4156388-supplementary.pdf]

cancers

MDPI

Supplementary Table S1. Summary of clinical, phenotypic, biochemical and cytogenetic data.

	**CD5+CD19+ cells [%]**	**CD19+CD5+CD23+ cells [%]**	**Serum IgA concentration [g/L]**	**Serum IgG concentration [g/L]**	**Serum IgM concentration [g/L]**	**Beta-2-microglobulin [mg/L]**	**Lactate dehydrogenase (LDH) [U/L]**
Mean	82.00	68.31	1.01	7.89	0.59	2.00	232.10
SD	7.50	8.30	0.72	2.67	0.46	0.47	78.19
Minimum	70.02	54.14	0.18	3.18	0.07	1.10	115.00
Maximum	98.41	79.99	2.93	14.50	1.58	3.02	466.00
Median	81.94	70.57	0.98	7.71	0.43	2.05	221.00
Q1	75.47	59.55	0.41	6.18	0.23	1.58	172.50
Q3	85.82	74.66	1.26	9.60	0.78	2.31	253.75

Supplementary Table S2. Summary table of demographic, clinical and biochemical data of all study subjects (patients and controls).

No	Variable	Stats / Values	Freqs (% of Valid)	Graph	Valid	Missing
1	Group [character]<br>1. CLL<br>2. Controls	30 (73.2%)<br>11 (26.8%)		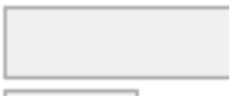	41 (100.0%)	0 (0.0%)
2	Age [numeric]<br>Mean (sd) : 71.2 (12.1)<br>min ≤ med ≤ max:<br>40 ≤ 75 ≤ 86<br>IQR (CV) : 16 (0.2)		24 distinct values	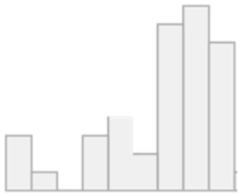	41 (100.0%)	0 (0.0%)
3	CLL_Classification [numeric]<br>Min : 0	0 : 16 (53.3%)<br>1 : 14 (46.7%)		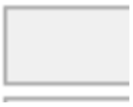	30 (73.2%)	11 (26.8%) (healthy controls)

 https://doi.org/10.3390/cancers18060896

	Mean : 0.5<br>Max : 1					
4	Gender [character]<br>1. M<br>2. W	13 (31.7%)<br>28 (68.3%)		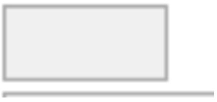	41 (100.0%)	0 (0.0%)
5	WBC [numeric]<br>Mean (sd) : 25.8 (15.6)<br>min ≤ med ≤ max:<br>4 ≤ 28.3 ≤ 47.9<br>IQR (CV) : 32.3 (0.6)		41 distinct values	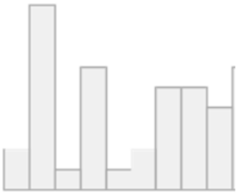	41 (100.0%)	0 (0.0%)
6	LYM [numeric]<br>Mean (sd) : 9 (6.4)<br>min ≤ med ≤ max:<br>0.8 ≤ 9.3 ≤ 35.6<br>IQR (CV) : 9.9 (0.7)		39 distinct values	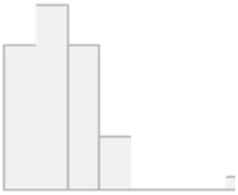	41 (100.0%)	0 (0.0%)
7	MON [numeric]<br>Mean (sd) : 0.6 (0.4)<br>min ≤ med ≤ max:<br>0.1 ≤ 0.5 ≤ 2<br>IQR (CV) : 0.2 (0.7)		32 distinct values	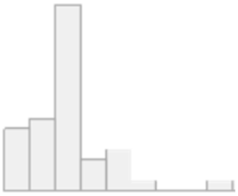	41 (100.0%)	0 (0.0%)
8	NEU [numeric]<br>Mean (sd) : 3.3 (1.3)<br>min ≤ med ≤ max:<br>0.6 ≤ 3.3 ≤ 6.3<br>IQR (CV) : 2 (0.4)		38 distinct values	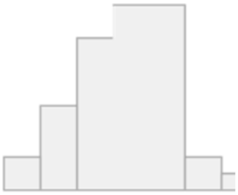	41 (100.0%)	0 (0.0%)
9	EOS [numeric]<br>Mean (sd) : 0.1 (0.1)<br>min ≤ med ≤ max:<br>0 ≤ 0.1 ≤ 0.5<br>IQR (CV) : 0.1 (0.7)		21 distinct values	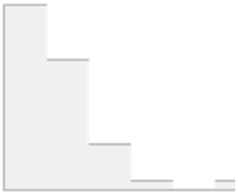	41 (100.0%)	0 (0.0%)

10	BAS [numeric]<br>Mean (sd) : 0.1 (0.1)<br>min ≤ med ≤ max:<br>0 ≤ 0 ≤ 0.2<br>IQR (CV) : 0.1 (0.8)		16 distinct values	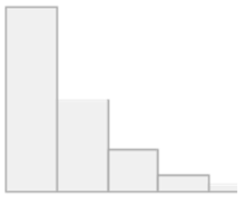	41 (100.0%)	0 (0.0%)
11	RBC [numeric]<br>Mean (sd) : 4.2 (0.7)<br>min ≤ med ≤ max:<br>2.3 ≤ 4.2 ≤ 5.2<br>IQR (CV) : 0.7 (0.2)		38 distinct values	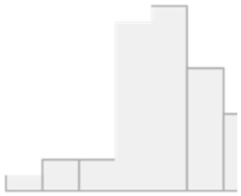	41 (100.0%)	0 (0.0%)
12	HGB [numeric]<br>Mean (sd) : 12.3 (1.8)<br>min ≤ med ≤ max:<br>8.2 ≤ 12.7 ≤ 15.5<br>IQR (CV) : 1.5 (0.1)		28 distinct values	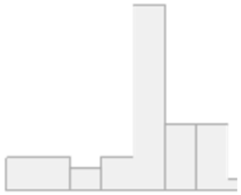	41 (100.0%)	0 (0.0%)
13	PLT [numeric]<br>Mean (sd) : 164 (83.6)<br>min ≤ med ≤ max:<br>34 ≤ 154 ≤ 404<br>IQR (CV) : 95 (0.5)		37 distinct values	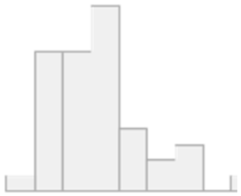	41 (100.0%)	0 (0.0%)
14	CD45 [numeric]<br>Mean (sd) : 96.5 (2.2)<br>min ≤ med ≤ max:<br>91.4 ≤ 97.3 ≤ 99.1<br>IQR (CV) : 3.4 (0)		38 distinct values	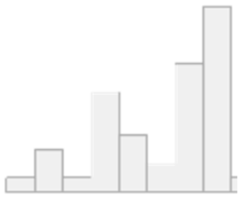	41 (100.0%)	0 (0.0%)
15	CD3 [numeric]<br>Mean (sd) : 32.9 (27.1)<br>min ≤ med ≤ max:<br>4.8 ≤ 18.4 ≤ 78.2<br>IQR (CV) : 55.9 (0.8)		41 distinct values	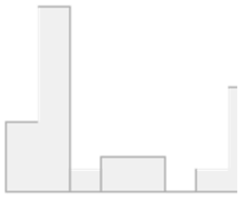	41 (100.0%)	0 (0.0%)

16	CD19 [numeric]<br>Mean (sd) : 59.5 (30)<br>min ≤ med ≤ max:<br>11.1 ≤ 75.8 ≤ 92.5<br>IQR (CV) : 66.7 (0.5)		41 distinct values	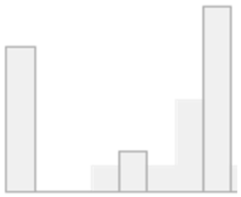	41 (100.0%)	0 (0.0%)
17	CD4 [numeric]<br>Mean (sd) : 21.3 (17.6)<br>min ≤ med ≤ max:<br>3 ≤ 11.2 ≤ 50.9<br>IQR (CV) : 34.7 (0.8)		41 distinct values	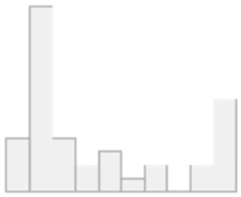	41 (100.0%)	0 (0.0%)
18	CD8 [numeric]<br>Mean (sd) : 12.2 (10.5)<br>min ≤ med ≤ max:<br>1.6 ≤ 6.3 ≤ 31.1<br>IQR (CV) : 19.7 (0.9)		41 distinct values	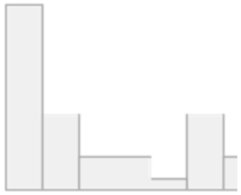	41 (100.0%)	0 (0.0%)
19	TC [numeric]<br>Mean (sd) : 247.9 (89.4)<br>min ≤ med ≤ max:<br>120 ≤ 257 ≤ 456.6<br>IQR (CV) : 142.5 (0.4)		41 distinct values	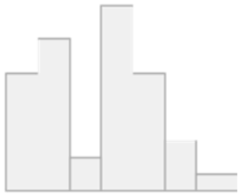	41 (100.0%)	0 (0.0%)
20	TG [numeric]<br>Mean (sd) : 151.6 (74.6)<br>min ≤ med ≤ max:<br>51.6 ≤ 135 ≤ 404<br>IQR (CV) : 95 (0.5)		39 distinct values	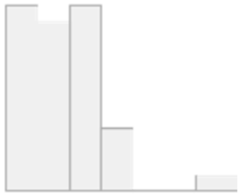	41 (100.0%)	0 (0.0%)

https://doi.org/10.3390/cancers18060896

21	HDL [numeric]<br>Mean (sd) : 62 (15.3)<br>min ≤ med ≤ max:<br>29.9 ≤ 63.5 ≤ 94<br>IQR (CV) : 19.7 (0.2)		37 distinct values	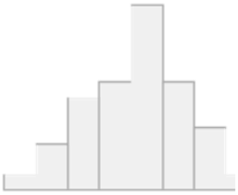	41 (100.0%)	0 (0.0%)
22	LDL [numeric]<br>Mean (sd) : 166 (63.2)<br>min ≤ med ≤ max:<br>70.9 ≤ 168 ≤ 338.5<br>IQR (CV) : 93 (0.4)		37 distinct values	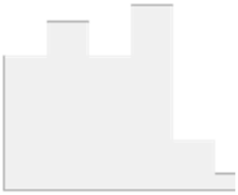	41 (100.0%)	0 (0.0%)
23	WBC_level [character]<br>1. normal<br>2. upper	11 (26.8%)<br>30 (73.2%)		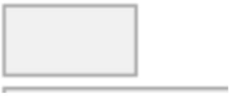	41 (100.0%)	0 (0.0%)
24	LYM_level [character]<br>1. normal<br>2. upper	11 (26.8%)<br>30 (73.2%)		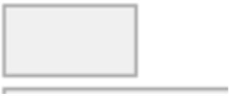	41 (100.0%)	0 (0.0%)
25	MON_level [character]<br>1. normal<br>2. upper	34 (82.9%)<br>7 (17.1%)		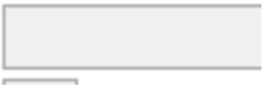	41 (100.0%)	0 (0.0%)
26	NEU_level [character]<br>1. lower<br>2. normal	4 (9.8%)<br>37 (90.2%)		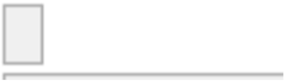	41 (100.0%)	0 (0.0%)
27	EOS_level [character]	1. normal<br>41 (100.0%)			41 (100.0%)	0 (0.0%)
28	BAS_level [character]<br>1. normal	40 (97.6%)<br>1 (2.4%)		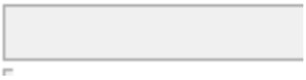	41 (100.0%)	0 (0.0%)

https://doi.org/10.3390/cancers18060896

	2. upper					
29	RBC_level [character]<br>1. lower<br>2. normal	28 (68.3%)<br>13 (31.7%)		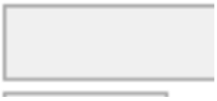	41 (100.0%)	0 (0.0%)
30	HGB_level [character]<br>1. lower<br>2. normal	27 (65.9%)<br>14 (34.1%)		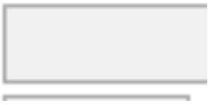	41 (100.0%)	0 (0.0%)
31	PLT_level [character]<br>1. lower<br>2. normal<br>3. upper	18 (43.9%)<br>22 (53.7%)<br>1 (2.4%)		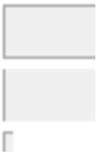	41 (100.0%)	0 (0.0%)
32	TC_level [character]<br>1. normal<br>2. upper	16 (39.0%)<br>25 (61.0%)		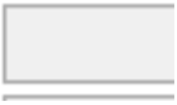	41 (100.0%)	0 (0.0%)
33	TG_level [character]<br>1. normal<br>2. upper	23 (56.1%)<br>18 (43.9%)		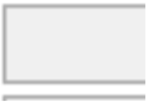	41 (100.0%)	0 (0.0%)
34	HDL_level [character]<br>1. lower<br>2. normal<br>3. upper	2 (4.9%)<br>34 (82.9%)<br>5 (12.2%)		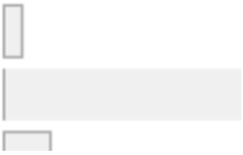	41 (100.0%)	0 (0.0%)
35	LDL_level [character]<br>1. normal<br>2. upper	15 (36.6%)<br>26 (63.4%)		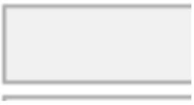	41 (100.0%)	0 (0.0%)

https://doi.org/10.3390/cancers18060896

Supplementary Table S3. Descriptive statistics and comparative analysis of aggregated lipid class abundances between treatment-naïve CLL patients and healthy controls.

Continuous_Variable	Subgroup_Variable	Group1	Group2	N1	N2	Median_Group1	Median_Group2	logFC	p_value	adj_p_value
CAR	group	CLL	Control	30	11	0,368376146	0,455164453	0,13488925	0,424013898	0,424013898
LPC	group	CLL	Control	30	11	14,81710219	10,48400485	-0,275634546	0,026822347	0,044703912
PC	group	CLL	Control	30	11	67,21835354	74,08979307	0,09664773	0,000214087	0,000535218
PC O-	group	CLL	Control	30	11	6,274426145	5,327560782	-0,272576379	0,000158609	0,000535218
SM	group	CLL	Control	30	11	11,46269835	10,29557927	-0,114255378	0,103068964	0,128836205

Supplementary Table X Legend: The table presents the median pseudo-abundances for each major lipid class (CAR, LPC, PC, PC O-, SM) aggregated across all individual species within the class. Statistical significance between treatment-naïve CLL patients (N=30) and healthy controls (N=11) was determined using the Wilcoxon-Mann-Whitney test. P-values were adjusted for multiple comparisons using the Benjamini-Hochberg (BH) false discovery rate method. (logFC: log2 pseudo-fold change; adj_p_value: Benjamini-Hochberg adjusted p-value).

Supplementary Table S4. DIFFERENTIAL EXPRESSION ANALYSIS.

feature	mean_exp	mean_ctrl	pval	padj	log2FC	A	negLogP	sig_fc.pval_color	Lipid.Maps.Category
CAR 10:0	0,009675651	0,006463572	0,026822347	0,116208629	0,582027132	-6,982439109	0,934761622	none	FA
CAR 10:1	0,00334928	0,000748026	0,203769859	0,407539718	2,162689977	-9,30327835	0,389830059	none	FA
CAR 10:2	0,000950893	0,000704127	0,850565476	0,941697491	0,43344754	-10,25515355	0,026088587	none	FA

https://doi.org/10.3390/cancers18060896

CAR 12:0	0,00358612	0,000765083	0,001083084	0,024142097	2,228735704	-9,237728354	1,617225009	up-regulated	FA
CAR 12:1	0,003365723	0,002997155	0,632151163	0,761036351	0,167323024	-8,298529159	0,118594599	none	FA
CAR 14:1	0,005199253	0,004411042	0,249181923	0,461172515	0,237184752	-7,706072372	0,336136584	none	FA
CAR 14:1;O	0,000501058	0,00044789	0,632151163	0,761036351	0,161832477	-11,0436516	0,118594599	none	FA
CAR 14:2	0,003079247	0,003717786	0,89623681	0,974854074	-0,271866232	-8,207273619	0,011060389	none	FA
CAR 16:0	0,007004714	0,005554123	0,44116509	0,601148035	0,334766956	-7,324841582	0,221018568	none	FA
CAR 16:1	0,001153345	0,001174845	0,390870674	0,576999567	-0,026646846	-9,74663726	0,238824513	none	FA
CAR 18:0	0,002356865	0,001709379	0,016233125	0,080516298	0,46339717	-8,960613559	1,0941162	none	FA
CAR 18:1	0,007345834	0,007623269	1	1	-0,053483481	-7,062116302	0	none	FA
CAR 18:1;O	0,000352673	0,000358633	0,57139641	0,738053696	-0,024175069	-11,45729314	0,13191204	none	FA
CAR 18:2	0,002266211	0,002000981	0,374888384	0,576999567	0,1795748	-8,875289579	0,238824513	none	FA
CAR 2:0	0,305717954	0,441797499	0,300998745	0,518386728	-0,531183971	-1,444134828	0,285346126	none	FA
CAR 3:0	0,021354918	0,019140003	0,407247435	0,594102141	0,157977293	-5,628276465	0,226138882	none	FA
CAR 3:1	0,000540193	0,00066181	0,390870674	0,576999567	-0,292941347	-10,70776656	0,238824513	none	FA

CAR 4:0	0,010682177	0,016154493	0,053019867	0,182623987	-0,596729845	-6,250285616	0,73844218	none	FA
CAR 4:0;O	0,002117725	0,003729892	0,116450411	0,283134334	-0,81661886	-8,47495981	0,548007464	none	FA
CAR 5:0	0,005221136	0,010453312	0,031433968	0,121806626	-1,001524526	-7,080658389	0,914329088	none	FA
CAR 5:1	0,003275116	0,005606503	0,014870677	0,07683183	-0,775555144	-7,866460546	1,11445882	none	FA
LPC 14:0	0,088209361	0,061993398	0,011351205	0,061197803	0,508817175	-3,757333018	1,213264166	none	GP
LPC 16:0	7,69562834	6,779141293	0,116450411	0,283134334	0,182936586	2,852570833	0,548007464	none	GP
LPC 16:1	0,165201776	0,151675447	0,344121087	0,554169024	0,123241629	-2,659319712	0,256357753	none	GP
LPC 17:0	0,122134704	0,085224357	0,004251439	0,032948653	0,519135488	-3,293022643	1,48216234	up-regulated	GP
LPC 18:0	2,600186121	1,833764791	0,001557564	0,024142097	0,503806292	1,126711749	1,617225009	up-regulated	GP
LPC 18:1	1,464069581	1,133574145	0,033975265	0,127664633	0,369105363	0,365431439	0,8939294	none	GP
LPC 18:2	1,452150705	0,928448309	0,005787676	0,037772201	0,645297689	0,21554234	1,42282771	up-regulated	GP
LPC 20:3	0,144116166	0,144719033	0,827901321	0,924862737	-0,006022495	-2,791684672	0,033922718	none	GP
LPC 20:4	0,436754052	0,379671653	0,13111354	0,301075535	0,202068801	-1,296141406	0,521324533	none	GP
LPC 24:0	0,012366481	0,012237628	0,988439471	1	0,015111191	-6,344976701	0	none	GP
LPC 26:0	0,018920489	0,028862538	0,17377981	0,365232144	-0,60924877	-5,419282391	0,437431007	none	GP

LPC 26:1	0,011487195	0,014600901	0,096838444	0,261042763	-0,346030924	-6,270814213	0,583288342	none	GP
LPC 28:0	0,015539402	0,019348325	0,214532828	0,415657355	-0,316277718	-5,849786366	0,38126453	none	GP
LPC 28:1	0,021427974	0,023070089	0,424013898	0,59747413	-0,106528163	-5,491096682	0,223680895	none	GP
PC 24:0	0,006140276	0,007744251	0,103068964	0,271926629	-0,334822183	-7,180069709	0,565548262	none	GP
PC 26:0	0,031477887	0,049398189	0,001083084	0,024142097	-0,650119435	-4,664457758	1,617225009	down-regulated	GP
PC 28:1	0,130114669	0,120737332	0,44116509	0,601148035	0,107911783	-2,996100372	0,221018568	none	GP
PC 30:0	0,151074781	0,122632871	0,079885524	0,2358525	0,30091711	-2,877123802	0,627359515	none	GP
PC 32:0	0,664773582	0,674604121	0,632151163	0,761036351	-0,021178079	-0,578476004	0,118594599	none	GP
PC 32:1	0,604863452	0,773612596	0,065358369	0,207806097	-0,355001794	-0,547817707	0,682341714	none	GP
PC 32:2	0,085254711	0,056368422	0,065358369	0,207806097	0,59689238	-3,85052282	0,682341714	none	GP
PC 32:3	0,014630118	0,013679665	0,374888384	0,576999567	0,096908487	-6,143369075	0,238824513	none	GP
PC 34:1	9,826556302	12,54930094	8,45068E-05	0,008433514	-0,35284918	3,473110505	2,073991409	down-regulated	GP
PC 34:2	16,76668986	18,97083517	0,022789059	0,108686283	-0,178185299	4,156618639	0,963825265	none	GP
PC 34:3	0,496975737	0,488442529	1	1	0,024986597	-1,021245974	0	none	GP

https://doi.org/10.3390/cancers18060896

PC 34:4	0,053849282	0,036266775	0,002206985	0,024878736	0,570278644	-4,500068403	1,604171692	up-regulated	GP
PC 36:0	0,050255871	0,020763319	0,003437003	0,030442026	1,275255058	-4,952191588	1,51652645	up-regulated	GP
PC 36:1	1,929888257	1,96583216	0,44116509	0,601148035	-0,026622836	0,961828734	0,221018568	none	GP
PC 36:2	9,417474058	8,389926326	0,029052157	0,116208629	0,166682012	3,151999148	0,934761622	none	GP
PC 36:3	4,9266946	5,331296945	0,513484806	0,6702328	-0,113866497	2,357553292	0,173774323	none	GP
PC 36:4	8,150447717	8,871333185	0,424013898	0,59747413	-0,122271618	3,08801512	0,223680895	none	GP
PC 36:5	0,935914008	0,736116258	0,214532828	0,415657355	0,346442345	-0,268773286	0,38126453	none	GP
PC 36:6	0,028904406	0,018575198	0,001557564	0,024142097	0,637911875	-5,4315227	1,617225009	up-regulated	GP
PC 38:0	0,106293134	0,078029555	0,005787676	0,037772201	0,445955831	-3,456857607	1,42282771	up-regulated	GP
PC 38:1	0,02728493	0,019387412	0,13111354	0,301075535	0,492984105	-5,442243874	0,521324533	none	GP
PC 38:3	1,900414856	1,974387645	0,738799324	0,864255813	-0,055090882	0,953859831	0,063357691	none	GP
PC 38:4	4,502210504	4,450590652	0,988439471	1	0,016636699	2,162315163	0	none	GP
PC 38:5	1,951337734	1,728664174	0,061014013	0,204479395	0,174805874	0,877060562	0,689350448	none	GP
PC 38:6	3,057920926	3,161890545	1	1	-0,048236326	1,636669264	0	none	GP
PC 40:1	0,015332956	0,016943214	0,261529776	0,469995539	-0,144071681	-5,955184462	0,327906264	none	GP

https://doi.org/10.3390/cancers18060896

PC 40:2	0,014826154	0,011204829	0,390870674	0,576999567	0,404023741	-6,277723689	0,238824513	none	GP
PC 40:3	0,023321148	0,017688923	0,029052157	0,116208629	0,3987926	-5,621613665	0,934761622	none	GP
PC 40:4	0,114044065	0,107045115	0,261529776	0,469995539	0,091372416	-3,178022928	0,327906264	none	GP
PC 40:5	0,317451597	0,309420716	0,873349887	0,958366247	0,036966846	-1,673874883	0,01846849	none	GP
PC 40:6	1,044480843	1,022712136	0,611603132	0,758387884	0,030385906	0,047593078	0,120108614	none	GP
PC 42:0	0,015424	0,013260931	0,287436841	0,502002371	0,217994808	-6,127676673	0,299294232	none	GP
PC 42:1	0,008357611	0,007177271	0,274280783	0,485868816	0,219655213	-7,012521216	0,313480973	none	GP
PC 42:2	0,008228695	0,007133725	0,12361781	0,294780932	0,206008161	-7,028124694	0,530500612	none	GP
PC 42:4	0,009151137	0,008389974	0,919207658	0,98260129	0,125284646	-6,834475572	0,00762267	none	GP
PC 42:5	0,012174137	0,010669382	0,237235209	0,452571783	0,19034292	-6,455208132	0,344312527	none	GP
PC 42:6	0,013003697	0,011252305	0,085255347	0,240265068	0,208701275	-6,36928495	0,619309366	none	GP
PC O-30:0	0,013666112	0,012054203	0,203769859	0,407539718	0,181066597	-6,283786584	0,389830059	none	GP
PC O-30:1	0,000910999	0,001951299	0,042635071	0,151049964	-1,098913144	-9,550805727	0,820879373	none	GP
PC O-30:2	0,004843303	0,004852174	0,988439471	1	-0,002639929	-7,688472962	0	none	GP

https://doi.org/10.3390/cancers18060896

PC O-32:1	0,107339019	0,089737578	0,138945516	0,313258982	0,258390448	-3,348948717	0,504096468	none	GP
PC O-32:2	0,026330924	0,023904737	0,359304085	0,571201366	0,139461596	-5,316828863	0,243210763	none	GP
PC O-34:0	0,049310266	0,044302501	0,424013898	0,59747413	0,154499871	-4,419218093	0,223680895	none	GP
PC O-34:1	0,395769373	0,375782451	0,591347523	0,755949411	0,074762278	-1,374649263	0,121507267	none	GP
PC O-34:2	0,425007509	0,302105934	0,001382249	0,024142097	0,492433808	-1,480656669	1,617225009	up-regulated	GP
PC O-34:3	0,232800187	0,160435064	0,005787676	0,037772201	0,537102727	-2,371387244	1,42282771	up-regulated	GP
PC O-36:0	0,026449478	0,027816654	0,476596444	0,642369121	-0,072709654	-5,204262116	0,192215344	none	GP
PC O-36:1	0,332950464	0,319435549	0,674071324	0,803700425	0,059782677	-1,616511883	0,094905802	none	GP
PC O-36:2	0,433852695	0,390019379	0,19339345	0,399679797	0,153659484	-1,281552545	0,398287804	none	GP
PC O-36:3	0,248680363	0,180207118	0,003083598	0,029412779	0,464636596	-2,239953802	1,531463939	up-regulated	GP
PC O-36:4	0,764021623	0,537384248	0,001752249	0,024142097	0,507659436	-0,642144344	1,617225009	up-regulated	GP
PC O-36:5	0,472134597	0,338212239	0,001752249	0,024142097	0,481269338	-1,32336456	1,617225009	up-regulated	GP
PC O-38:0	0,071188588	0,055895406	0,010348953	0,05833046	0,348916258	-3,986668339	1,2341046	none	GP
PC O-38:1	0,033563233	0,030027554	0,919207658	0,98260129	0,160594718	-4,977271868	0,00762267	none	GP

PC O-38:2	0,078110441	0,066386654	0,32934147	0,54451123	0,234622157	-3,795651871	0,263993159	none	GP
PC O-38:3	0,237812009	0,211992989	0,988439471	1	0,165805021	-2,155009034	0	none	GP
PC O-38:4	0,496485536	0,400765358	0,009423591	0,055644059	0,308993883	-1,164673347	1,254581201	none	GP
PC O-38:5	0,707670926	0,525826421	0,001968067	0,024404028	0,428492014	-0,713095453	1,612538494	up-regulated	GP
PC O-38:6	0,274991856	0,191106092	0,000136024	0,008433514	0,525015122	-2,125046764	2,073991409	up-regulated	GP
PC O-40:1	0,043372102	0,032612163	0,00639708	0,039661894	0,411357269	-4,732767446	1,401626549	up-regulated	GP
PC O-40:2	0,0772125	0,06681278	0,249181923	0,461172515	0,208710335	-3,79937693	0,336136584	none	GP
PC O-40:3	0,095606735	0,079568413	0,942244263	0,998617852	0,264916439	-3,519202155	0,000600675	none	GP
PC O-40:4	0,110105401	0,088930571	0,109603571	0,277364139	0,308133897	-3,337109801	0,556949691	none	GP
PC O-40:5	0,174655771	0,151135435	0,390870674	0,576999567	0,208672364	-2,621749962	0,238824513	none	GP
PC O-40:6	0,141391352	0,115865766	0,039567796	0,144306079	0,287239509	-2,965853971	0,840715374	none	GP
PC O-42:0	0,019147388	0,020841445	0,17377981	0,365232144	-0,122307661	-5,645554731	0,437431007	none	GP
PC O-42:1	0,014254033	0,012672164	0,344121087	0,554169024	0,169707219	-6,217339641	0,256357753	none	GP
PC O-42:2	0,018135643	0,014890062	0,026822347	0,116208629	0,284478114	-5,927267388	0,934761622	none	GP

PC O-42:3	0,023776671	0,020545286	0,17377981	0,365232144	0,210739362	-5,499679126	0,437431007	none	GP
PC O-42:4	0,029153564	0,025689527	0,147121019	0,32576797	0,182491928	-5,191429879	0,487091618	none	GP
PC O-42:5	0,087048737	0,082902918	0,827901321	0,924862737	0,070400485	-3,557233066	0,033922718	none	GP
PC O-44:3	0,004772872	0,004405017	0,760807601	0,881683575	0,115709998	-7,768781591	0,05468725	none	GP
PC O-44:4	0,009573053	0,009783679	0,783005117	0,899005875	-0,031397915	-6,691106225	0,04623747	none	GP
PC O-44:5	0,048749792	0,048913667	0,738799324	0,864255813	-0,00484154	-4,356039346	0,063357691	none	GP
PC O-44:6	0,033611127	0,030819935	0,611603132	0,758387884	0,125075099	-4,957454837	0,120108614	none	GP
SM 18:1;O2/14:1;O	0,240900524	0,203133026	0,090903374	0,250489298	0,246012712	-2,17649692	0,601210824	none	SP
SM 18:1;O2/16:0	5,424226744	4,781396123	0,074785327	0,226180015	0,181985554	2,34842471	0,645545772	none	SP
SM 18:1;O2/16:1	0,685405706	0,598858503	0,109603571	0,277364139	0,194743035	-0,64234141	0,556949691	none	SP
SM 18:1;O2/16:1;O	0,130749544	0,116209314	0,314967125	0,527782751	0,170080208	-3,020162283	0,277544808	none	SP
SM 18:1;O2/18:0	0,988121942	1,08329595	0,314967125	0,527782751	-0,132666435	0,049094215	0,277544808	none	SP
SM 18:1;O2/18:1	0,440325388	0,43943891	0,827901321	0,924862737	0,002907409	-1,184811771	0,033922718	none	SP
SM 18:1;O2/20:2	0,010746027	0,011474279	0,513484806	0,6702328	-0,094600069	-6,492752731	0,173774323	none	SP

https://doi.org/10.3390/cancers18060896

SM 18:1;O2/22:1;O	0,425093199	0,355688157	0,029052157	0,116208629	0,25716624	-1,362732037	0,934761622	none	SP
SM 18:1;O2/22:2;O	0,387264359	0,300957292	0,002471031	0,025533992	0,36375996	-1,550489342	1,592881282	up-regulated	SP
SM 18:1;O2/24:0	0,631829286	0,575806642	0,085255347	0,240265068	0,133950378	-0,729368474	0,619309366	none	SP
SM 18:1;O2/24:1	2,151625666	2,100602576	0,513484806	0,6702328	0,034623867	1,08811517	0,173774323	none	SP
SM 18:1;O2/24:1;O	0,039394038	0,033626414	0,074785327	0,226180015	0,228382373	-4,780070089	0,645545772	none	SP
SM 18:1;O2/26:0	0,005471781	0,004190045	0,004251439	0,032948653	0,385044732	-7,706296272	1,48216234	up-regulated	SP
SM 18:1;O2/26:1	0,013863425	0,012866252	0,611603132	0,758387884	0,107691874	-6,226418384	0,120108614	none	SP

Supplementary Table S5. THE FILTERED SIGNIFICANT RESULTS.

feature	mean_ctrl	mean_exp	sd_ctrl	sd_exp	FC	log2FC	method	statistic	pval	negLog10pval	padj	negLog10padj	sig_pval	sig_padj
PC 34:1	12,5493	9,826556	2,365285	1,366569	0,783036	-0,35285	Wilcoxon test	40	8,45068E-05	4,073108377	0,008433514	2,073991409	yes	yes
PC O-38:6	0,191106	0,274992	0,044694	0,081712	1,438949	0,525015	Wilcoxon test	287	0,000136024	3,866383099	0,008433514	2,073991409	yes	yes
CAR 12:0	0,000765	0,003586	0,001882	0,001816	4,68723	2,228736	Wilcoxon test	272	0,001083084	2,965337696	0,024142097	1,617225009	yes	yes
LPC 18:0	1,833765	2,600186	0,646838	0,516984	1,41795	0,503806	Wilcoxon test	269	0,001557564	2,807554119	0,024142097	1,617225009	yes	yes
PC 26:0	0,049398	0,031478	0,016209	0,011422	0,637228	-0,65012	Wilcoxon test	58	0,001083084	2,965337696	0,024142097	1,617225009	yes	yes
PC 36:6	0,018575	0,028904	0,005239	0,011422	1,556075	0,637912	Wilcoxon test	269	0,001557564	2,807554119	0,024142097	1,617225009	yes	yes
PC O-34:2	0,302106	0,425008	0,093373	0,11114	1,406816	0,492434	Wilcoxon test	270	0,001382249	2,859413631	0,024142097	1,617225009	yes	yes

https://doi.org/10.3390/cancers18060896

PC O-36:4	0,537384	0,764022	0,101952	0,224068	1,421742	0,507659	Wilcoxon test	268	0,001752249	2,756404185	0,024142097	1,617225009	yes	yes
PC O-36:5	0,338212	0,472135	0,072835	0,126409	1,395971	0,481269	Wilcoxon test	268	0,001752249	2,756404185	0,024142097	1,617225009	yes	yes
PC O-38:5	0,525826	0,707671	0,100241	0,165236	1,345826	0,428492	Wilcoxon test	267	0,001968067	2,705960179	0,024404028	1,612538494	yes	yes
PC 34:4	0,036267	0,053849	0,00781	0,018822	1,48481	0,570279	Wilcoxon test	266	0,002206985	2,656200692	0,024878736	1,604171692	yes	yes
SM 18:1;O2/22:2;O	0,300957	0,387264	0,085058	0,060251	1,286775	0,36376	Wilcoxon test	265	0,002471031	2,607121721	0,025533992	1,592881282	yes	yes
PC O-36:3	0,180207	0,24868	0,041712	0,070651	1,37997	0,464637	Wilcoxon test	263	0,003083598	2,510942272	0,029412779	1,531463939	yes	yes
PC 36:0	0,020763	0,050256	0,019025	0,031959	2,420416	1,275255	Wilcoxon test	262	0,003437003	2,463820099	0,030442026	1,51652645	yes	yes
LPC 17:0	0,085224	0,122135	0,026796	0,033329	1,433096	0,519135	Wilcoxon test	260	0,004251439	2,371464042	0,032948653	1,48216234	yes	yes
SM 18:1;O2/26:0	0,00419	0,005472	0,000834	0,00161	1,3059	0,385045	Wilcoxon test	260	0,004251439	2,371464042	0,032948653	1,48216234	yes	yes
LPC 18:2	0,928448	1,452151	0,510423	0,478608	1,564062	0,645298	Wilcoxon test	257	0,005787676	2,237495794	0,037772201	1,42282771	yes	yes
PC 38:0	0,07803	0,106293	0,018715	0,036695	1,362216	0,445956	Wilcoxon test	257	0,005787676	2,237495794	0,037772201	1,42282771	yes	yes
PC O-34:3	0,160435	0,2328	0,071329	0,055346	1,451056	0,537103	Wilcoxon test	257	0,005787676	2,237495794	0,037772201	1,42282771	yes	yes
PC O-40:1	0,032612	0,043372	0,009944	0,008671	1,329936	0,411357	Wilcoxon test	256	0,00639708	2,194018239	0,039661894	1,401626549	yes	yes

Supplementary Table S6. FEATURE IMPORTANCE AND SIGNAL-TO-NOISE METRICS OF THE STABLE LIPID DISCRIMINATORS IDENTIFIED BY THE BORUTA ALGORITHM.

feature_label	medianImp	Ratio_to_ShadowMax	Ratio_to_ShadowMean
CAR 12:0	0.0799984942406016	0.92640466195391	25,38
LPC 18:0	0.0716972803904596	0.830274312456096	22,74
LPC 16:0	0.0695268765029518	0.805140435890083	22,06
CAR 10:0	0.0659497194568141	0.763715969148042	20,92
PC O-36:5	0.0650596240245607	0.753408418164101	20,64
LPC 18:1	0.063429514795653	0.734531302994754	20,12
LPC 18:2	0.0620741435211873	0.71883572923099	19,69

Supplementary Table Y. Legend: The table presents the raw importance metrics for the seven robust lipid biomarkers distinguishing treatment-naïve CLL patients from healthy controls. Feature selection was executed using the Boruta algorithm coupled with the Random Ferns importance measure. Median Importance (Z-score) reflects the median importance generated across all algorithm iterations. Signal-to-noise separation is quantified by the Ratio to Maximum Shadow (the lipid's median importance divided by the highest importance score achieved by any permuted shadow attribute) and the Ratio to Mean Shadow (the lipid's median importance divided by the average importance score of all shadow attributes). The high ratio values confirm robust signal separation from randomized background noise for all identified lipid discriminators.

Supplementary Table S7. RECEIVER OPERATING CHARACTERISTIC (ROC) ANALYSIS METRICS FOR CANDIDATE LIPID BIOMARKERS IN CHRONIC LYMPHOCYTIC LEUKEMIA.

Lipid	AUC	CI_Lower	CI_Upper	threshold	sensitivity	specificity
CAR 12:0	0,824242424	0,641481293	1	0,000288384	0,9	0,909090909
LPC 18:0	0,815151515	0,639595313	0,990707717	2,203892892	0,833333333	0,818181818
PC O-36:5	0,812121212	0,681644196	0,942598228	0,441193254	0,633333333	1
LPC 18:2	0,778787879	0,580086271	0,977489487	1,066296402	0,8	0,818181818
CAR 10:0	0,727272727	0,556194873	0,898350581	0,009287055	0,566666667	0,818181818
LPC 18:1	0,718181818	0,500835445	0,935528192	1,106541319	0,833333333	0,636363636
LPC 16:0	0,663636364	0,455674811	0,871597916	6,418792348	0,8	0,545454545

Supplementary Table Z Legend: The table details the diagnostic performance of the seven stable lipid discriminators differentiating treatment-naïve CLL patients from healthy controls. Diagnostic accuracy was quantified using the Area Under the Curve (AUC) alongside 95% confidence intervals (CI). The optimal classification threshold for each lipid species, as well as its corresponding sensitivity and specificity, was determined by maximizing Youden's J statistic.

Supplementary Table S8. GENE FUNCTIONS (GATOm- Gene Annotation Tool for Metabolomics).

Lipid	Log2FC	p-value
CAR 12:0	2,228736	0,001083
PC 36:0	1,275255	0,003437
LPC 18:2	0,645298	0,005788
LPC 18:2	0,645298	0,005788
PC 36:6	0,637912	0,001558
CAR 10:0	0,582027	0,026822
PC 34:4	0,570279	0,002207
LPC 17:0	0,519135	0,004251

https://doi.org/10.3390/cancers18060896

LPC 14:0	0,508817	0,011351
PC O-36:4	0,507659	0,001752
LPC 18:0	0,503806	0,001558
PC O-36:5	0,481269	0,001752
CAR 18:0	0,463397	0,016233
SM 18:1;O2/26:0	0,385045	0,004251
LPC 18:1	0,369105	0,033975
LPC 18:1	0,369105	0,033975
PC O-38:4	0,308994	0,009424
PC 36:2	0,166682	0,029052
PC 34:2	-0,17819	0,022789
PC 34:1	-0,35285	8,45E-05
PC 34:1	-0,35285	8,45E-05
PC 26:0	-0,65012	0,001083
PC 26:0	-0,65012	0,001083
Carnicor		
sn-glycerol 3-phosphocholine		
phosphocholine		
tetradecanoate		
(5Z,8Z,11Z,14Z)-eicosatetraenoate		
1,2-ditetradecanoyl-sn-glycero-3-phosphocholine		
(5Z,8Z,11Z,14Z)-eicosatetraenoyl-CoA		
decanoyl-CoA		
1-hexadecanoyl-sn-glycero-3-phosphocholine		
1-O-octadecyl-sn-glycero-3-phosphocholine		
1-heptadecanoyl-2-(5Z,8Z,11Z,14Z-eicosatetraenoyl)-sn-glycero-3-phosphocholine		

Supplementary Table S9. THE ASSOCIATED LIPIDS FOR EACH GENE (GATOm).

Path	Reaction	Gene
PC 26:0 -> LPC 18:0	1-octadecanoyl-2-octanoyl-sn-glycero-3-phosphocholine + H2O = 1-octadecanoyl-sn-glycero-3-phosphocholine + H(+) + octanoate	ABHD3
Carnicor -> CAR 10:0	(R)-carnitine + decanoyl-CoA = CoA + O-decanoyl-(R)-carnitine	CPT2
Carnicor -> CAR 12:0	(R)-carnitine + dodecanoyl-CoA = CoA + O-dodecanoyl-R-carnitine	CPT2
Carnicor -> CAR 18:0	(R)-carnitine + octadecanoyl-CoA = CoA + O-octadecanoyl-(R)-carnitine	CPT2
LPC 18:1 -> PC 34:1	1-(9Z-octadecenoyl)-sn-glycero-3-phosphocholine + hexadecanoyl-CoA = 1-(9Z-octadecenoyl)-2-hexadecanoyl-sn-glycero-3-phosphocholine + CoA	LPCAT3
LPC 18:1 -> PC 36:2	(9Z)-octadecenoyl-CoA + 1-(9Z-octadecenoyl)-sn-glycero-3-phosphocholine = 1,2-di-(9Z-octadecenoyl)-sn-glycero-3-phosphocholine + CoA	LPCAT3
sn-glycerol 3-phosphocholine -> LPC 18:1	1-(9Z-octadecenoyl)-sn-glycero-3-phosphocholine + H2O = (9Z)-octadecenoate + H(+) + sn-glycerol 3-phosphocholine	PNPLA6

https://doi.org/10.3390/cancers18060896

LPC 18:1 -> phosphocholine	1-(9Z-octadecenoyl)-sn-glycero-3-phosphocholine + H2O = 1-(9Z-octadecenoyl)-sn-glycerol + H(+) + phosphocholine	ENPP6
LPC 18:2 -> phosphocholine	1-(9Z,12Z)-octadecadienoyl-sn-glycero-3-phosphocholine + H2O = 1-(9Z,12Z-octadecadienoyl)-sn-glycerol + H(+) + phosphocholine	ENPP6
tetradecanoate -> 1,2-ditetradecanoyl-sn-glycero-3-phosphocholine	1,2-ditetradecanoyl-sn-glycero-3-phosphocholine + H2O = 1-tetradecanoyl-sn-glycero-3-phosphocholine + H(+) + tetradecanoate	ABHD3
1,2-ditetradecanoyl-sn-glycero-3-phosphocholine -> LPC 14:0	1,2-ditetradecanoyl-sn-glycero-3-phosphocholine + H2O = 1-tetradecanoyl-sn-glycero-3-phosphocholine + H(+) + tetradecanoate	ABHD3
(5Z,8Z,11Z,14Z)-eicosatetraenoate -> PC O-36:4	1-O-hexadecyl-2-(5Z,8Z,11Z,14Z)-eicosatetraenoyl-sn-glycero-3-phosphocholine + H2O = (5Z,8Z,11Z,14Z)-eicosatetraenoate + 1-O-hexadecyl-sn-glycero-3-phosphocholine + H(+)	PLA2G4F
PC O-36:4 -> (5Z,8Z,11Z,14Z)-eicosatetraenoyl-CoA	(5Z,8Z,11Z,14Z)-eicosatetraenoyl-CoA + 1-O-hexadecyl-sn-glycero-3-phosphocholine = 1-O-hexadecyl-2-(5Z,8Z,11Z,14Z)-eicosatetraenoyl-sn-glycero-3-phosphocholine + CoA	LPCAT4
(5Z,8Z,11Z,14Z)-eicosatetraenoyl-CoA -> PC O-38:4	(5Z,8Z,11Z,14Z)-eicosatetraenoyl-CoA + 1-O-octadecyl-sn-glycero-3-phosphocholine = 1-O-octadecyl-2-(5Z,8Z,11Z,14Z)-eicosatetraenoyl-sn-glycero-3-phosphocholine + CoA	LPCAT2
CAR 10:0 -> decanoyl-CoA	(R)-carnitine + decanoyl-CoA = CoA + O-decanoyl-(R)-carnitine	CPT2
decanoyl-CoA -> PC 26:0	1-hexadecanoyl-sn-glycero-3-phosphocholine + decanoyl-CoA = 1-hexadecanoyl-2-decanoyl-sn-glycero-3-phosphocholine + CoA	LPCAT1
phosphocholine -> LPC 14:0	1-tetradecanoyl-sn-glycero-3-phosphocholine + H2O = 1-tetradecanoyl-sn-glycerol + H(+) + phosphocholine	ENPP6
phosphocholine -> SM 18:1;O2/26:0	an N-(acyl)-sphingosylphosphocholine + H2O = an N-acyl-sphingoid base + H(+) + phosphocholine	SMPD3
1-hexadecanoyl-sn-glycero-3-phosphocholine -> PC 34:1	(9Z)-octadecenoyl-CoA + 1-hexadecanoyl-sn-glycero-3-phosphocholine = 1-hexadecanoyl-2-(9Z-octadecenoyl)-sn-glycero-3-phosphocholine + CoA	LPCAT3
1-hexadecanoyl-sn-glycero-3-phosphocholine -> PC 34:2	(9Z,12Z)-octadecadienoyl-CoA + 1-hexadecanoyl-sn-glycero-3-phosphocholine = 1-hexadecanoyl-2-(9Z,12Z-octadecadienoyl)-sn-glycero-3-phosphocholine + CoA	LPCAT3
1-hexadecanoyl-sn-glycero-3-phosphocholine -> PC 26:0	1-hexadecanoyl-sn-glycero-3-phosphocholine + decanoyl-CoA = 1-hexadecanoyl-2-decanoyl-sn-glycero-3-phosphocholine + CoA	LPCAT1
1-hexadecanoyl-sn-glycero-3-	1-hexadecanoyl-sn-glycero-3-phosphocholine + eicosanoyl-CoA = 1-hexadecanoyl-2-eicosanoyl-sn-glycero-3-phosphocholine + CoA	LPCAT1

phosphocholine -> PC 36:0		
PC 34:1 -> LPC 18:1	1-hexadecanoyl-2-(9Z-octadecenoyl)-sn-glycero-3-phosphocholine + N-(acetyl)-sphing-4-enine = 1-hexadecanoyl-N-(acetyl)-sphing-4-enine + 2-(9Z-octadecenoyl)-sn-glycero-3-phosphocholine	PLA2G15
PC 34:2 -> LPC 18:2	1-hexadecanoyl-2-(9Z,12Z-octadecadienoyl)-sn-glycero-3-phosphocholine + N-(acetyl)-sphing-4-enine = 1-hexadecanoyl-N-(acetyl)-sphing-4-enine + 2-(9Z,12Z-octadecadienoyl)-sn-glycero-3-phosphocholine	PLA2G15
sn-glycerol 3-phosphocholine -> LPC 18:0	1-octadecanoyl-sn-glycero-3-phosphocholine + H2O = H(+) + octadecanoate + sn-glycerol 3-phosphocholine	LYPLA2
PC 36:2 -> LPC 18:1	1,2-di-(9Z-octadecenoyl)-sn-glycero-3-phosphocholine + H2O = (9Z)-octadecenoate + 2-(9Z-octadecenoyl)-sn-glycero-3-phosphocholine + H(+)	PLAAT3
1-O-octadecyl-sn-glycero-3-phosphocholine -> PC O-38:4	(5Z,8Z,11Z,14Z)-eicosatetraenoyl-CoA + 1-O-octadecyl-sn-glycero-3-phosphocholine = 1-O-octadecyl-2-(5Z,8Z,11Z,14Z)-eicosatetraenoyl-sn-glycero-3-phosphocholine + CoA	LPCAT2
phosphocholine -> 1-O-octadecyl-sn-glycero-3-phosphocholine	1-O-octadecyl-sn-glycero-3-phosphocholine + H2O = 1-O-octadecyl-sn-glycerol + H(+) + phosphocholine	SMPD3
(5Z,8Z,11Z,14Z)-eicosatetraenoate -> PC O-36:5	1-O-(1Z)-hexadecenyl-2 (5Z,8Z,11Z,14Z)-eicosatetraenoyl-sn-glycero-3-phosphocholine + H2O = (5Z,8Z,11Z,14Z)-eicosatetraenoate + 1-(1Z-hexadecenyl)-sn-glycero-3-phosphocholine + H(+)	PNPLA8
(5Z,8Z,11Z,14Z)-eicosatetraenoate -> 1-heptadecanoyl-2-(5Z,8Z,11Z,14Z-eicosatetraenoyl)-sn-glycero-3-phosphocholine	1-heptadecanoyl-2-(5Z,8Z,11Z,14Z-eicosatetraenoyl)-sn-glycero-3-phosphocholine + H2O = (5Z,8Z,11Z,14Z)-eicosatetraenoate + 1-heptadecanoyl-sn-glycero-3-phosphocholine + H(+)	ABHD16A
LPC 17:0 -> 1-heptadecanoyl-2-(5Z,8Z,11Z,14Z-eicosatetraenoyl)-sn-glycero-3-phosphocholine	1-heptadecanoyl-2-(5Z,8Z,11Z,14Z-eicosatetraenoyl)-sn-glycero-3-phosphocholine + H2O = (5Z,8Z,11Z,14Z)-eicosatetraenoate + 1-heptadecanoyl-sn-glycero-3-phosphocholine + H(+)	ABHD16A
tetradecanoate -> PC 34:4	1-tetradecanoyl-2-(5Z,8Z,11Z,14Z-eicosatetraenoyl)-sn-glycero-3-phosphocholine + H2O = 2-(5Z,8Z,11Z,14Z)-eicosatetraenoyl-sn-glycero-3-phosphocholine + H(+) + tetradecanoate	ABHD3
tetradecanoate -> PC 36:6	1-tetradecanoyl-2-(4Z,7Z,10Z,13Z,16Z,19Z-docosahexaenoyl)-sn-glycero-3-phosphocholine + H2O = 2-(4Z,7Z,10Z,13Z,16Z,19Z-docosahexaenoyl)-sn-glycero-3-phosphocholine + H(+) + tetradecanoate	ABHD3

Supplementary Table S10. THE MOST SIGNIFICANTLY ENRICHED BIOLOGICAL PROCESSES RELATED TO LIPID METABOLISM.

GO	Category	Description	Count	%	Log10(P)	Log10(q)
GO:0046486	GO Biological Processes	glycerolipid metabolic process	13	86.67	-23.23	-19.16
GO:0046470	GO Biological Processes	phosphatidylcholine metabolic process	9	60.00	-20.26	-16.68
R-HSA-556833	Reactome Gene Sets	Metabolism of lipids	13	86.67	-19.39	-15.89
GO:0016042	GO Biological Processes	lipid catabolic process	11	73.33	-19.00	-15.57
hsa00565	KEGG Pathway	Ether lipid metabolism	6	40.00	-13.13	-10.00
GO:0006658	GO Biological Processes	phosphatidylserine metabolic process	4	26.67	-9.55	-6.59
GO:0006631	GO Biological Processes	fatty acid metabolic process	6	40.00	-8.16	-5.26
GO:0006639	GO Biological Processes	acylglycerol metabolic process	4	26.67	-6.86	-4.08
GO:0015909	GO Biological Processes	long-chain fatty acid transport	3	20.00	-5.60	-2.88

https://doi.org/10.3390/cancers18060896

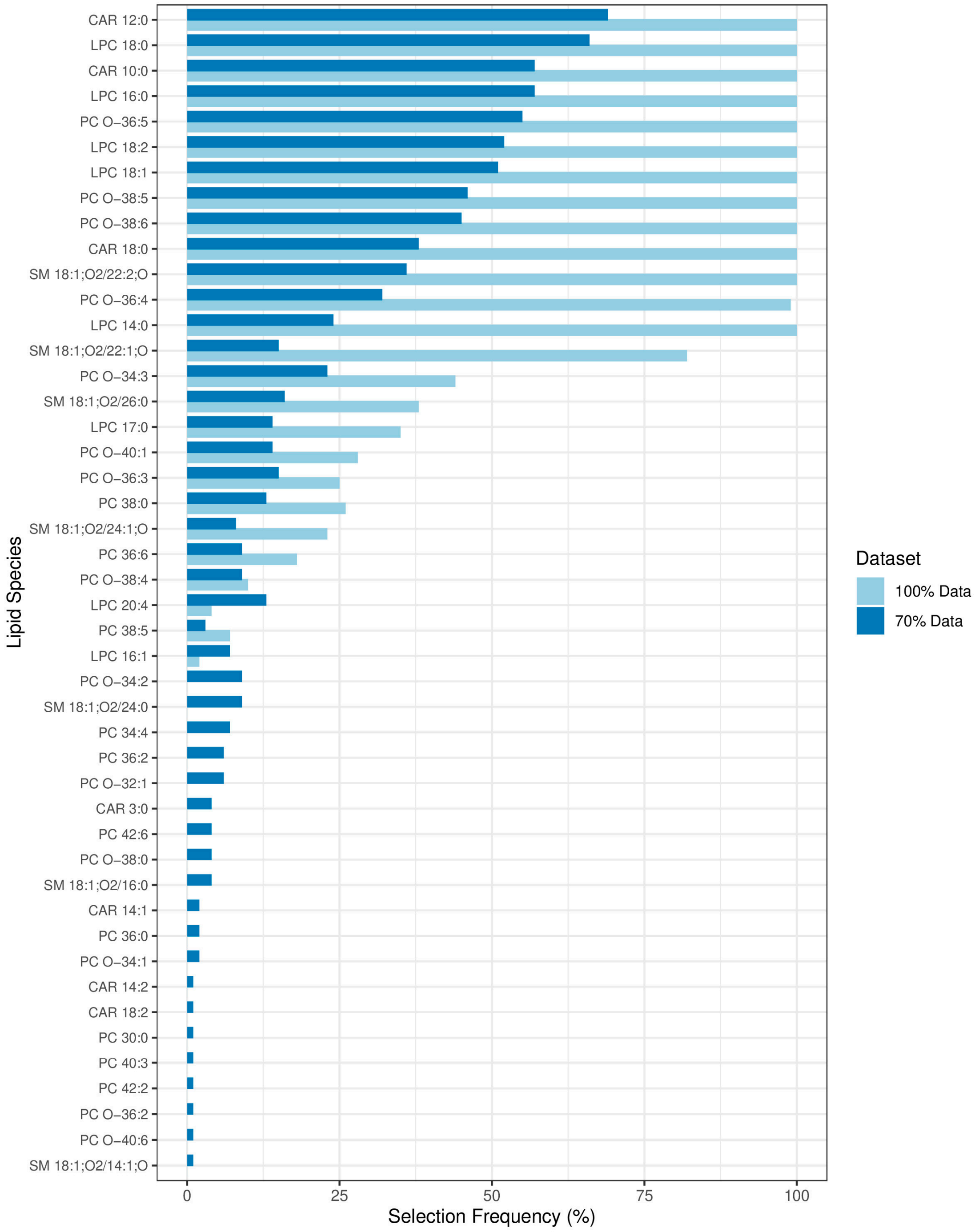

Figure S1. Feature selection analysis using the Boruta algorithm identified lipids that distinguish CLL patients from controls.

https://doi.org/10.3390/cancers18060896

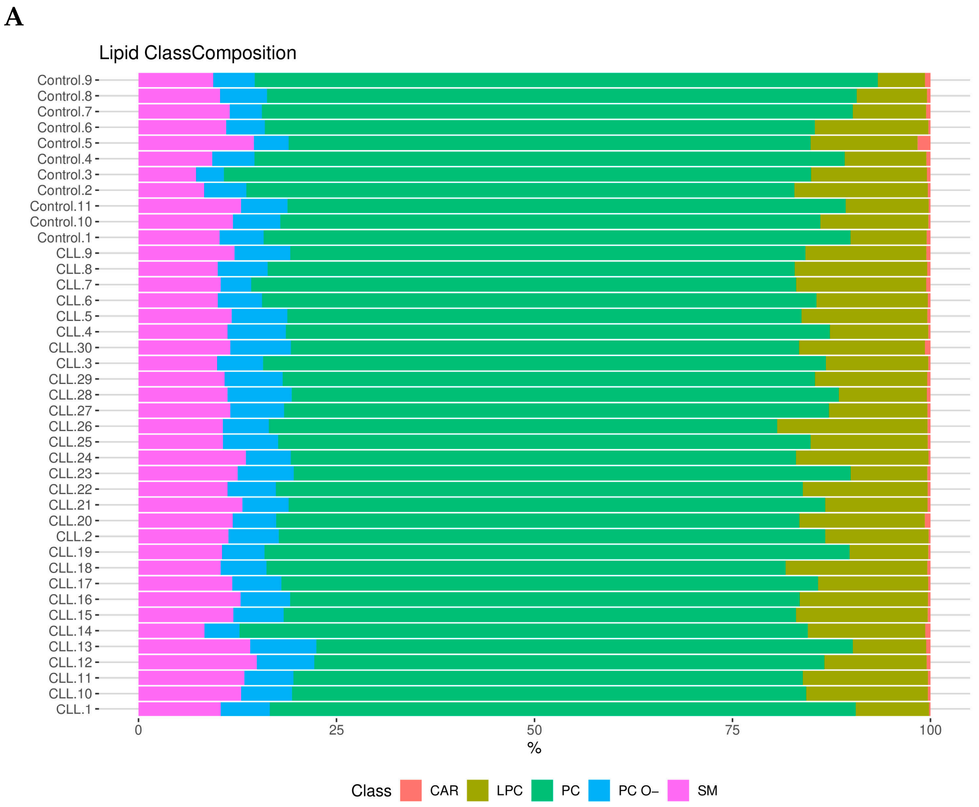

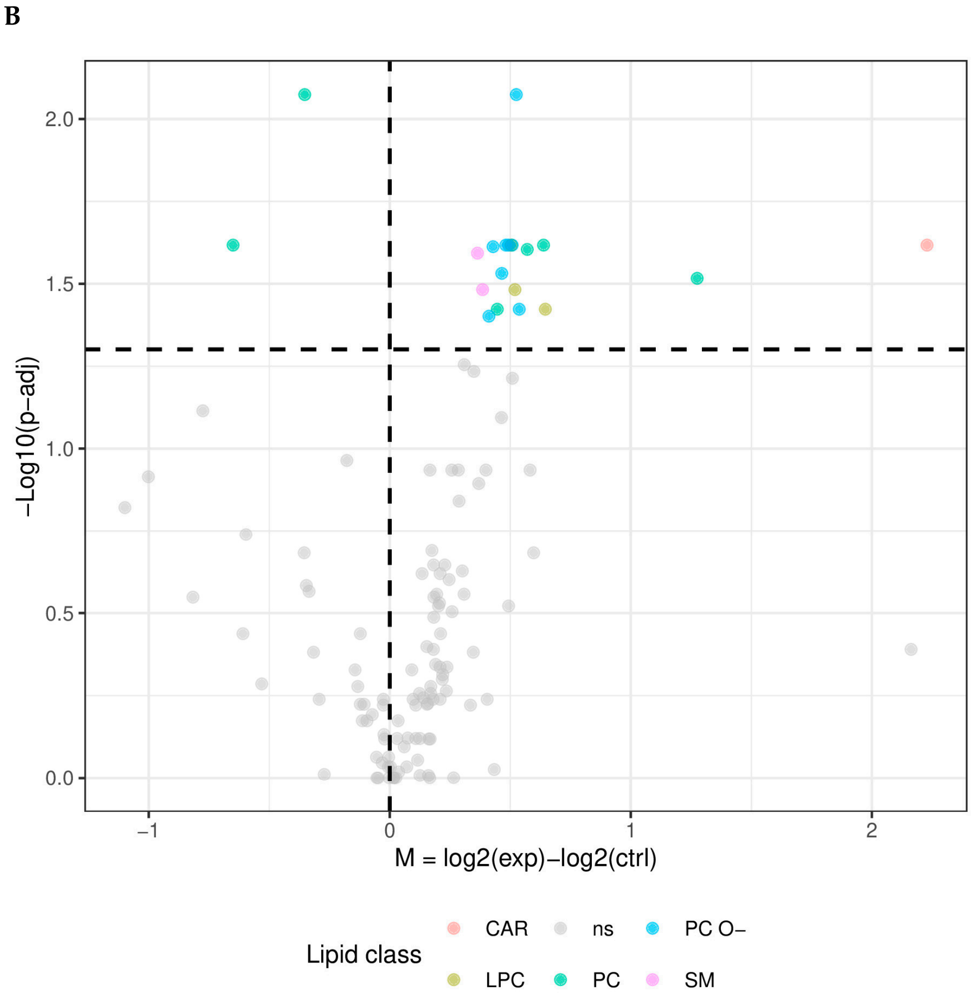

Figure S2. Lipid composition (A) and differential expression analysis (B) between CLL and controls.

https://doi.org/10.3390/cancers18060896
